# Machine-learning-derived predictive score for early estimation of COVID-19 mortality risk in hospitalized patients

**DOI:** 10.1371/journal.pone.0274171

**Published:** 2022-09-22

**Authors:** Alba González-Cebrián, Joan Borràs-Ferrís, Juan Pablo Ordovás-Baines, Marta Hermenegildo-Caudevilla, Mónica Climente-Marti, Sonia Tarazona, Raffaele Vitale, Daniel Palací-López, Jesús Francisco Sierra-Sánchez, Javier Saez de la Fuente, Alberto Ferrer

**Affiliations:** 1 Multivariate Statistical Engineering Group, Department of Applied Statistics and Operational Research and Quality, Universitat Politècnica de València, València, España; 2 Pharmacy Service, Hospital Universitario Dr. Peset, València, España; 3 Univ. Lille, CNRS, LASIRE - UMR 8516 - Laboratory of Advanced Spectroscopy for Interaction, Reactivity and Environmental Studies, Lille, France; 4 Pharmacy Service, Hospital Universitario Jerez de la Frontera, Área de Gestión Sanitaria Jerez-Costa Noroeste y Sierra de Cádiz, Jerez de la Frontera, España; 5 Pharmacy Service, Hospital Universitario Ramón y Cajal, Madrid, España; Hospital Universitat de Sant Joan, Universitat Rovira I Virgili, SPAIN

## Abstract

The clinical course of COVID-19 is highly variable. It is therefore essential to predict as early and accurately as possible the severity level of the disease in a COVID-19 patient who is admitted to the hospital. This means identifying the contributing factors of mortality and developing an easy-to-use score that could enable a fast assessment of the mortality risk using only information recorded at the hospitalization. A large database of adult patients with a confirmed diagnosis of COVID-19 (n = 15,628; with 2,846 deceased) admitted to Spanish hospitals between December 2019 and July 2020 was analyzed. By means of multiple machine learning algorithms, we developed models that could accurately predict their mortality. We used the information about classifiers’ performance metrics and about importance and coherence among the predictors to define a mortality score that can be easily calculated using a minimal number of mortality predictors and yielded accurate estimates of the patient severity status. The optimal predictive model encompassed five predictors (age, oxygen saturation, platelets, lactate dehydrogenase, and creatinine) and yielded a satisfactory classification of survived and deceased patients (area under the curve: 0.8454 with validation set). These five predictors were additionally used to define a mortality score for COVID-19 patients at their hospitalization. This score is not only easy to calculate but also to interpret since it ranges from zero to eight, along with a linear increase in the mortality risk from 0% to 80%. A simple risk score based on five commonly available clinical variables of adult COVID-19 patients admitted to hospital is able to accurately discriminate their mortality probability, and its interpretation is straightforward and useful.

## Introduction

The pandemic produced by the SARS-CoV-2 virus in 2020–2022 has caused to date (Aug 2022) more than 560 million infections and more than six million deaths worldwide, already ranking in many countries as one of the three main causes of death. In Spain, one of the European countries most affected by this pandemic, there have been more than 13 million infections and more than 109,000 deaths [1].

The clinical course of COVID-19 is highly variable, and although most infected patients suffer minor flu symptoms, 10%—20% of them require hospitalization, (mainly due to the development of bilateral pneumonia, and hypoxemia), and 10–15% of these develop a serious respiratory illness requiring mechanical ventilation or ICU admission, which increases the risk of death [2]. Progression to severe disease appears to be linked to damages to organs other than the respiratory tract that occur through an organic inflammatory syndrome possibly related to massive cytokines release [3].

In the clinical setting, it is essential to predict the severity level of the disease in a COVID-19 patient who is admitted to the hospital, both from the individual point of view and for what concerns potential health system collapses, whose prevention requires decisions about patient management with appropriate triage criteria. This prediction involves identifying the contributing factors of mortality, which enables the adoption of targeted strategies in high-risk patients [4]. Most of the therapies (monoclonal antibodies, remdesivir, molnupiravir, specific protease inhibitors, etc.) that could improve the prognosis of this disease are useful applied early, within the first days after the appearance of symptoms. Therefore, an early identification of the risk of death from COVID-19 can be critical.

Several groups of researchers have published observational prognostic studies on COVID-19 patients so as to identify predictive variables of death or severity of illness. However, later works have highlighted the need of a clearer statistical assessment on these type of studies, ensuring the statistical coherence and the prevention of bias in finally proposed models [5, 6].

The objective of this study are i) to determine key predictors of mortality in adult patients admitted to the hospital with a diagnosis of SARS-CoV-2 infection, ii) to obtain a predictive model of mortality for these patients, and iii) to propose a reliable and easy-to-use mortality risk score that can be calculated readily and straightforwardly at hospital admission.

## Materials and methods

### Setting and patients

The data used in this study were obtained from the RERFAR-COVID-19-SEFH Registry, a nationwide prospective registry sponsored by the Spanish Society of Hospital Pharmacy (SEFH). It is a big repository of anonymized COVID-19 medical records of 15,628 patients admitted to Spanish hospitals from March 20th to July 15th, 2020. The study protocol was approved by the Spanish Agency for Medicines and Medical Devices (AEMPS) and the Institutional Review Boards of the 174 participating hospitals. The protocol is available online at the European Network of Centers for Pharmacoepidemiology and Pharmacovigilance (ENCePP)(R) website [7].

All registered patients were diagnosed using SARS-CoV-2 testing on nasopharyngeal swabs (real-time reverse transcriptase-polymerase chain reaction) at the time of admission. Data were collected and managed using REDCap electronic data capture tools hosted at SEFH [8]. This huge database contained up to 256 fields for each patient from patient admission to death or 42 days following hospital discharge. A total number of 1,036 pharmacists from 174 hospitals contributed to the collection of anonymized data from the patients’ electronic medical records.

In order to prevent over-representation bias arising from large hospitals, a maximum of 200 patients per hospital were recommended. Patient selection was carried out by centralized randomization up to 200 patients in each hospital.

### Outcomes and variables

The primary endpoint was all-cause mortality, codified as the binary variable “mortality” with, levels “alive” (numerically as zero) or “deceased” (numerically as one). Baseline was the date of hospital admission. The follow-up censoring date was July 15th, 2020; if a patient had not reached the outcome (death) by the time the data were obtained, their outcome was considered as null. Clinical routine data from medical records available in the database included demographic variables, clinical conditions at admission, comorbidities (type and number), chronic medication treatments, biochemical and hematological analytics, and timing of events (from the onset of symptoms to emergency room visit, admission or microbiological diagnosis)—see Table 1.

**Table 1:** Blocks of variables included in the data set registered at the admission event of a patient with COVID-19.

Block	Number of variables	Variable Names
Demographic variables	2	Age, Sex.
Clinical variables at admission	6	Fever within previous 24h (Fever 24), Conscience, Respiratory frequency > 24 breaths per minute (Rf 24), Systolic Blood Pressure < 90 mmHg within the previous 24 hours (SBP 90), Affected quadrants, Oxygen saturation.
Comorbidities	11	High Blood Pressure (HBP), Diabetes Mellitus (DM), Chronic Obstructive Pulmonary Disease (COPD), Asthma, Cardiac Failure, Ischemic Heart Disease (IHD), Kidney failure, Cirrhosis, Neurological precedents, Neoplasia, Number of comorbidities
Pharmacological treatments for chronic conditions	4	Previous treatment with ACEI, ARB, Previous treatment with NSAID, Previous treatment with montelukast.
Analytics at admission	12	Creatinine, Lactate dehydrogenase (LDH), Leukocytes, Neutrophils, Lymphocytes, Platelets, C-reactive protein (CRP), Hemoglobin, Procalcitonin (PCT), Neutrophils to Lymphocytes Ratio (NLR), Lymphocytes to CRP Ratio (LCR), Platelets to Lymphocytes Ratio (PLR).
Admission event variables	3	Time between the initial symptoms and the arrival to emergency room (Time init—urg), Time between the initial symptoms and the hospital admission (Time init—admission), Time between the initial symptoms and the microbiological confirmation (Time init—micro).

ACEI—angiotensin converting enzyme inhibitors; ARB—Angiotensin II receptor blockers; NSAID—Non-steroidal anti-inflammatory drugs.

### Statistical analysis methodology

**Fig 1:**
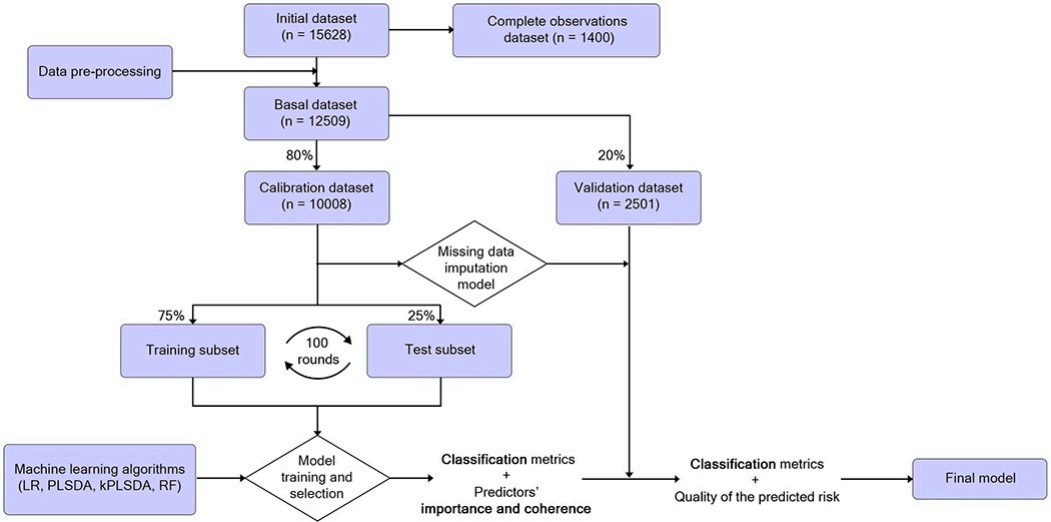
Flux diagram of the data used for the mortality prediction model building and validation. Data were stored in the REDCap storing service. The initial database (n = 15,628) was preprocessed and split into a calibration (n = 10,008) and validation (n = 2,501) subsets, without replacement. The calibration data set was used to set the optimal hyperparameters of the classifiers. The final model was chosen assessing the performance with the validation data set. LR = Logistic Regression. PLSDA = Partial Least Squares—Discriminant Analysis. kPLSDA = kernel PLSDA. RF = Random Forest.

The work aimed to build a model that, provided a set of variables recorded at the hospital admission, could predict the mortality risk of a patient with COVID-19 during admission and until 42 days following hospital discharge. The methodology for model training, evaluation and comparison is illustrated in Fig 1.

The initial data set (n = 15,628 with 2,846 deceased individuals) was preprocessed to obtain a clean Basal data set (n = 12,509). This depuration process eliminated variables and observations with excessive missing values or errors in the data. A preliminary univariate study (Table 2 and S1 Table) was conducted to explore potential significant predictors for the mortality outcome. This way, the missing data percentage could be reduced while still being cautious of keeping potentially important predictors. Technical details of these steps are described in depth in the S1 File (pp 1–6).

**Table 2:** Characteristics of patients in the complete data set.

	Complete data set				
	Total; *n* = 1400	Alive; *n* = 1243	Deceased; *n* = 157	Odds ratio	*p*value
**Variable**	m(s.d.) or n(%)	m(s.d.) or n(%)	m(s.d.) or n(%)	(95%C.I.)	
*Age, years*	63.82 (14.73)	62.44 (14.44)	74.75 (12.27)	1.07 (1.06;1.09)	<0.0001
*Oxygen saturation, %*	92.9 (5.5)	93.36 (5.1)	89.27 (7.01)	0.91 (0.88;0.93)	<0.0001
*Platelets*, 10^3^/*mm*^3^	208.63 (87.55)	210.14 (88)	196.66 (83.21)	1 (1;1)	0.07
*LDH, U/L*	375.17 (193.31)	362.29 (178.78)	477.14 (262.37)	1 (1;1)	<0.0001
*Creatinine, mg/dl*	1.01 (0.64)	0.97 (0.57)	1.34 (1.01)	1.73 (1.52;1.93)	<0.0001
*Lymphocytes*, 10^3^/*mm*^3^	1.68 (4.18)	1.73 (4.36)	1.3 (2.27)	0.95 (0.86;1.03)	0.21
*Leukocytes*, 10^3^/*mm*^3^	7.34 (5.21)	7.17 (5.14)	8.65 (5.59)	1.04 (1.01;1.06)	0.006
*Hemoglobin*, 10^3^/*mm*^3^	13.89 (1.95)	13.92 (1.94)	13.6 (2)	0.92 (0.83;1)	0.047
*D dimer*, 10^3^/*mm*^3^	1,233.45 (2,488.68)	1,092.22 (2,017.04)	2,351.62 (4,662.09)	1 (1;1)	<0.0001
*Time init—admission, days*	7.14 (4.83)	7.27 (4.64)	6.08 (6.07)	0.94 (0.89;0.98)	0.0028
*N. of comorbidities*	1.22 (1.23)	1.13 (1.2)	1.92 (1.31)	1.57 (1.44;1.69)	<0.0001
*Altered conscience*				6.09 (5.62;6.55)	<0.0001
*No*	1,312 (93.71%)	1,189 (95.66%)	123 (78.34%)		
*Yes*	88 (6.29%)	54 (4.34%)	34 (21.66%)		
*Respiratory frequency > 24 bpm*				2.58 (2.24;2.92)	<0.0001
*No*	1,028 (73.43%)	942 (75.78%)	86 (54.78%)		
*Yes*	372 (26.57%)	301 (24.22%)	71 (45.22%)		
*Cardiac failure*				2.15 (1.52;2.79)	0.018
*No*	1,337 (95.5%)	1,193 (95.98%)	144 (91.72%)		
*Yes*	63 (4.5%)	50 (4.02%)	13 (8.28%)		
*Neurological precedents*				2.46 (2.05;2.86)	<0.0001
*No*	1,219 (87.07%)	1,100 (88.5%)	119 (75.8%)		
*Yes*	181 (12.93%)	143 (11.5%)	38 (24.2%)		
*Neoplasia*				1.31 (0.7;1.92)	0.38
*No*	1,307 (93.36%)	1,163 (93.56%)	144 (91.72%)		
*Yes*	93 (6.64%)	80 (6.44%)	13 (8.28%)		
*SBP < 90*				4.09 (3.61;4.58)	<0.0001
*No*	1,313 (93.79%)	1,183 (95.17%)	130 (82.8%)		
*Yes*	87 (6.21%)	60 (4.83%)	27 (17.2%)		
*Kidney failure*				2.8 (2.28;3.32)	0.00012
*No*	1,314 (93.86%)	1,178 (94.77%)	136 (86.62%)		
*Yes*	86 (6.14%)	65 (5.23%)	21 (13.38%)		

Summary of the univariate tests based on the odds-ratio yielded by univariate logistic regression models built between every predictor and the mortality response. p-values in bold are < 1.10^−6^. For each numerical predictor, the mean and the standard deviation (in parentheses) values are indicated. For each categorical predictor, the number, and the percentage (in parentheses) of cases are reported.

Afterwards, as seen in Fig 1, the basal data set was randomly split into the calibration data set (n = 10,008) and the validation data set, (n = 2,501). The calibration data set was used to fit classifiers and to build a missing data imputation model using an adaptation of the Trimmed Scores Regression method (S1 File p 6). The imputed Calibration data set was repeatedly (100 repetitions) split in a training subset and a test subset. Four supervised algorithmic techniques were used as classifiers: Logistic Regression (LR) [9], Partial Least Squares Discriminant Analysis (PLSDA) [10], kernel-PLSDA (kPLSDA) [11], and Random Forest (RF) [12].

In each repetition of the calibration, all classifiers were trained and then used to predict the mortality of the test subset. Next, all classifiers were compared in three different types of assessment:

- Assessment of the classification performance. S4 Table describes the metrics used to evaluate the performance of the models for the classification of survival and deceased individuals.
- Assessment of the importance and coherence of the measured variables:
  1. The importance coefficient of each variable was calculated over the 100 repetitions. These coefficients were calculated differently for each classification algorithm. A more detailed explanation about the calculation of these coefficients for each model can be found in S1 File (p 6). A set of variables’ importance coefficients was obtained, informing about both the magnitude of the predictor’s importance and the sign of its relation to the mortality: positive coefficients are interpreted as risk factors and negative coefficients denote protection factors against the mortality by COVID-19.
  2. Using the predictor importance coefficients, all the 38 predictors were ranked according to the magnitudes of their corresponding coefficients. The coherence of the sign for each coefficient was also calculated as the percentage of folds in which the sign was kept the same. Predictors with a sign coherence below 75%, were eliminated from subsequent steps.
  3. Then, an incremental strategy was followed using the set of most important and coherent predictors. It consisted in the sequential construction of classification models encompassing a progressively larger number of predictors. The predictors were sorted according to the previous importance ranking. Thus, the performance of the model was tracked along the inclusion of gradually less important predictors. This approach enabled the identification of the minimal set of the most important variables recorded at hospital admission that were linked to COVID-19 mortality.
- Assessment of the quality of risk calibration. An assessment of the quality of the prediction risk was also performed as proposed in [4]. The underlying idea is that the assigned label (alive or deceased) is chosen according to the predicted probability of pertaining to the corresponding class, i.e., the risk of mortality in this case. This is critical in medical problems where, in fact, decisions may be based on the associated risk, and not directly on the final label. With this purpose, a calibration curve is fitted between the predicted risk (x-axis) and the observed proportion of deceased patients for that level of predicted risk (y-axis). In our case, we divided the predicted risk (ranging from zero to one), into groups separated by a step of 0.1. The perfect risk calibration would have a zero intercept term and a slope of one, having a death rate of 10% for the group of patients exhibiting a mortality risk of 0.1, a death rate of 20% for the group of patients exhibiting a mortality risk of 0.2, and so on. This way, progressively higher predicted risk values would be associated to higher mortality proportions within the studied sample.

The optimal model was selected considering the model that, with the minimum number of most important predictors, yielded the best classification performance and the best calibration of the predicted risk [13]. Finally, the information about the optimal model was used to configure the mortality score model. The idea of this procedure is to try to replicate a simplified classifier which is not based on a complicated and device-based calculus.

All data sets are accessible in ZENODO [14]. The statistical analysis was executed using MATLAB (2020b), R 4.0.2, and Python 3.8.3.

## Results

The first part of this section reports a descriptive analysis. Secondly, the results obtained by the four classifiers to predict the mortality are presented and compared. Finally, the confection of the mortality score based on the structure of the best model, is explained.

An initial univariate analysis was done to identify the variables that could be potentially important in further steps of the study. Such analysis was done, first, with the data set of complete observations. Table 2 shows the results only for those predictors that were found to be the most relevant *a posteriori*, based on the results obtained by the four classification techniques exploited in this study. S1 Table shows the results for non-relevant predictors from Table 1 that are not included in Table 2. Moreover, univariate analysis were also carried out on the imputed Calibration and Validation data sets, to check the coherence of the results from Table 2. S2 and S3 Tables contain the results for the Calibration and the Validation data sets, respectively, considering the variables from Table 2, and showing coherence between all three data sets.

According to the preliminary analysis shown in Table 2, deceased patients presented higher levels of the following risk factors at hospital admission: age, creatinine levels, SBP under 90 and respiratory frequency above 24 bpm. Deceased patients also exhibited a higher proportion of altered conscience. Besides, the number of comorbidities (≥2) was also significantly higher in deceased individuals, with a higher prevalence of cardiac failure, neurological antecedents, neoplasia, or kidney failure. On the contrary, deceased patients presented significantly lower values for the following protective factors: oxygen saturation, platelets and lymphocytes at hospital admission.

Next, we evaluated the different classification models for predicting COVID-19 outcome by using the framework of repeated training and testing. The best performance was achieved by the Random Forest classifier, with a median AUC of 0.8648, but results were very similar for all the methods when including all predictors (S1 Fig and S5 Table). However, as reducing the number of features eases the practical implementation of a classifier, we implemented a forward step-wise approach to select the minimum number of predictors for the final model, while maintaining the trade-off between performance and usability.

**Fig 2:**
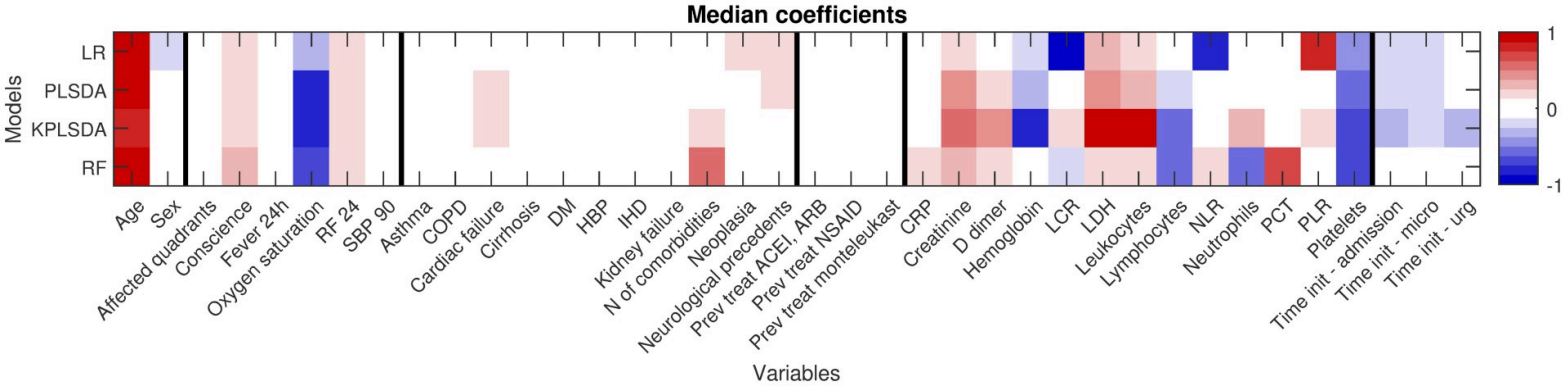
Importance metrics for all predictors. Median values (over the 100 re-sampling folds) of the 38 predictor coefficients sorted by type of data blocks (demographic variables, clinical variables at admission, comorbidities, pharmacological treatments for chronic conditions, analytics at admission and information about the admission event).

Using the predictor importance coefficients, all the 38 predictors were ranked according to the values of their corresponding coefficients. Fig 2 displays the median predictor coefficients over the 100 re-sampling folds. Color intensity indicates the strength of the relation between each predictor and the mortality risk. Positive coefficients are represented in red and denote risk factors (positively correlated with the mortality risk), while negative coefficients are graphed in blue and connote protection factors against mortality by COVID-19.

**Fig 3:**
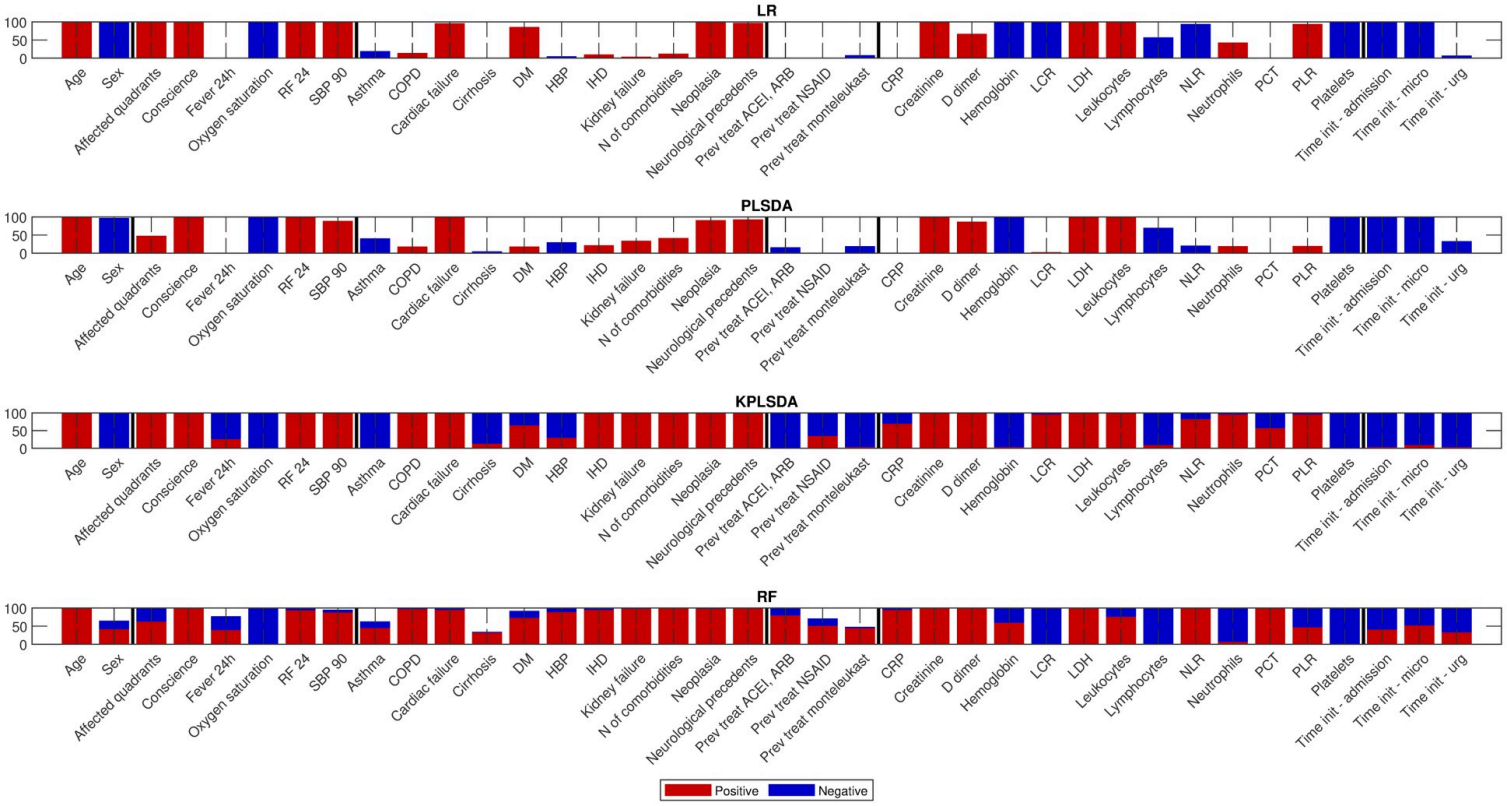
Coherence metrics for all predictors and classifiers. Bar charts representing the percentage of folds in which each predictor was found to show a positive (red) or a negative coefficient (blue) for the LR model (A), the PLSDA model (B), the kPLSDA model (C), and the RF model (D). Bars with high color consistency indicate highly consistent relationships between predictors and mortality.

The coefficient sign consistency over the aforementioned 100 folds, instead can be inferred from Fig 3, where each bar indicates the percentage of times the corresponding coefficient was found to be positive or negative. Low consistency points out unclear relationships with the mortality risk that may simply arise from the adopted re-sampling scheme and might not be necessarily substantiated by biomedical rationales.

**Fig 4:**
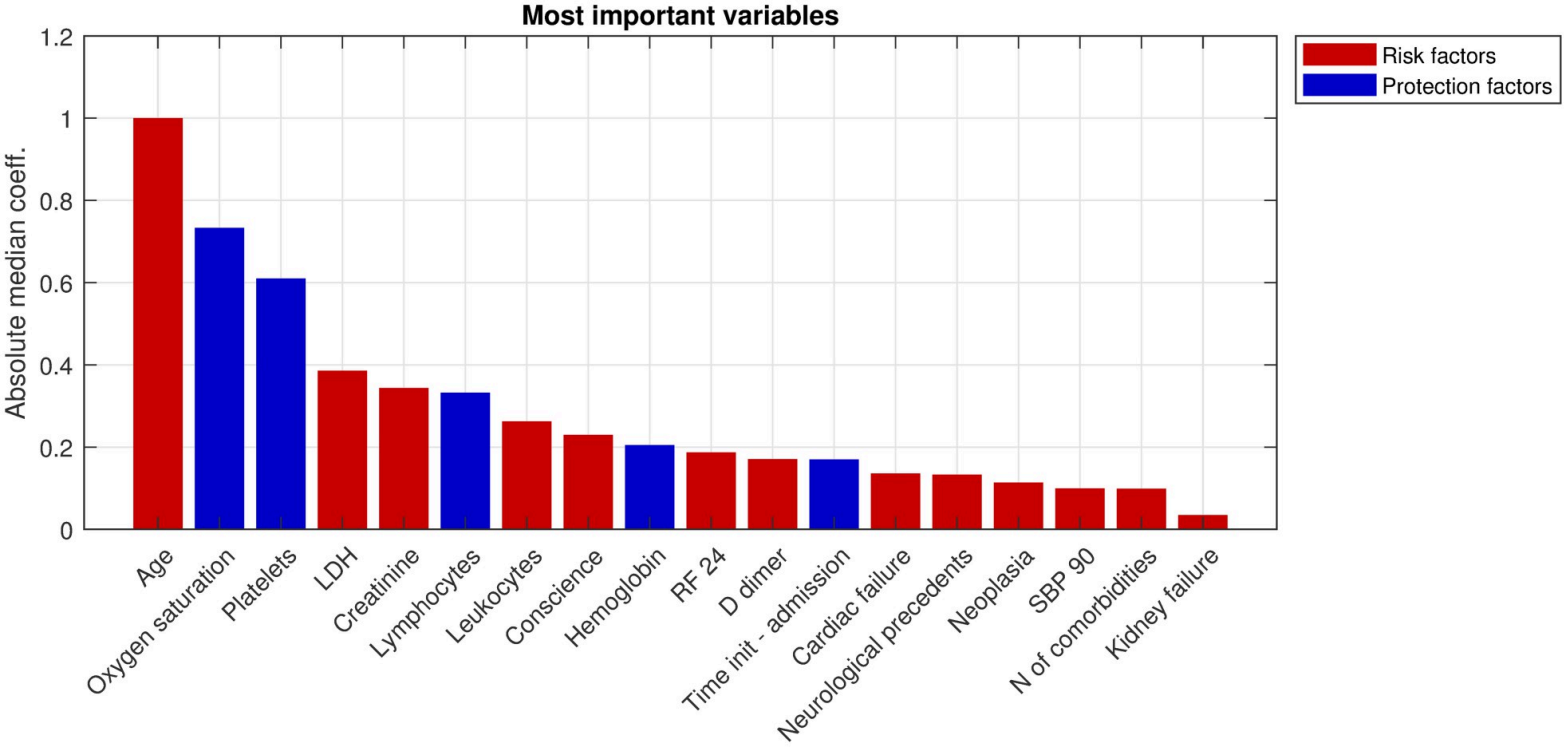
Importance of most relevant variables. Ranking (in descending order) of the 18 variables selected according to their importance and to the consistency of their relationship with the mortality risk over the 100 re-sampling iterations.

Based on both the magnitude of the coefficients and the consistency of their sign, a subset of 18 features showing high median coefficient (absolute) values and high sign consistency (above 75%), was selected. Fig 4 shows the absolute value of their median coefficients, sorted in descending order.

**Fig 5:**
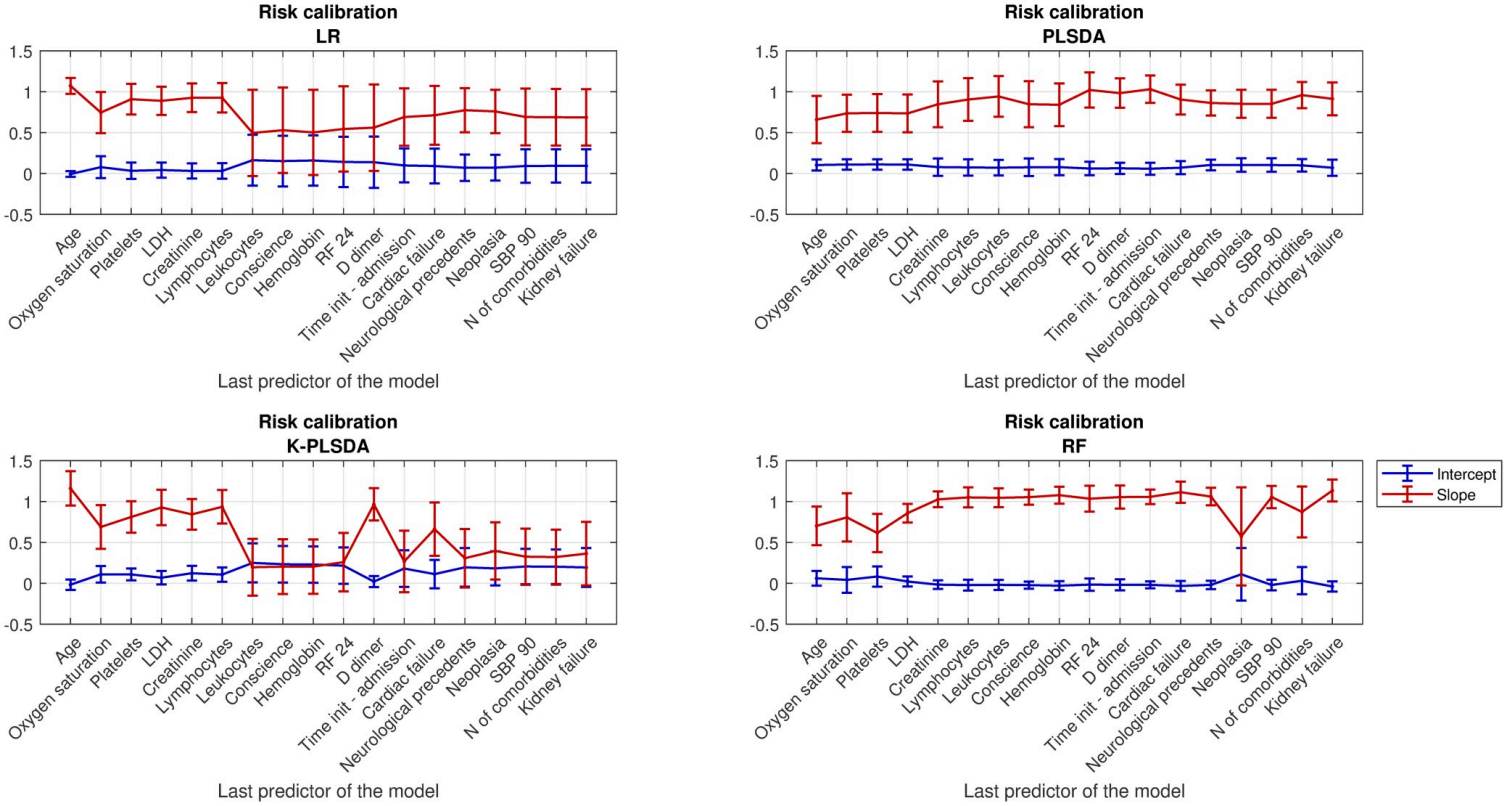
Assessment on the quality of the risk calibration. Intercept and slope of the risk calibration curve obtained for each incremental model with LR (A), PLSDA (B), kPLSDA (C) and RF (D).

The ranking of the most important features was finally used for the sake of model validation. To this end, an incremental strategy was implemented. A first model to predict mortality was fitted using only the most important feature (age) and its corresponding classification metrics were obtained. Afterwards, the second most predictive feature (oxygen saturation), was additionally considered for model calibration and the resulting classification metrics were stored. This was iterated for all 18 features from Fig 5.

The results obtained when the trained models were used to classify the validation data set, were assessed from two different perspectives. In first place, the metrics from S4 Table were calculated to report the overall classification performance. The results (S2 Fig) showed that satisfactory classification metrics were achieved by means of all the employed classifiers. The LR, RF and kPLSDA classifiers yielded an AUC around 0.85 with the final validation data set. Besides, their evolution with the number of important variables modeled seemed to be in strong agreement. This coherence was a good indicator of the overall classification quality, but it was important to account for another criterion for the determination of the best classifier: the quality of the prediction risk.

A second assessment on the quality of the predicted risk completed the performance report. This type of analysis is especially relevant for medical classification models, given the direct implications that an over (or under) estimation of the mortality risk can have in the medical decision-making. With this purpose a calibration curve was fitted using the information about the predicted risk (x-axis) and the observed proportion of deceased patients among those within that group of predicted risk (y-axis) [13]. Fig 5 shows the estimated intercept and slope of the risk calibration curve fitted for each incremental model and classification technique. Confidence intervals were calculated assuming a confidence level of 95% and using the standard error of the estimated coefficients.

**Fig 6:**
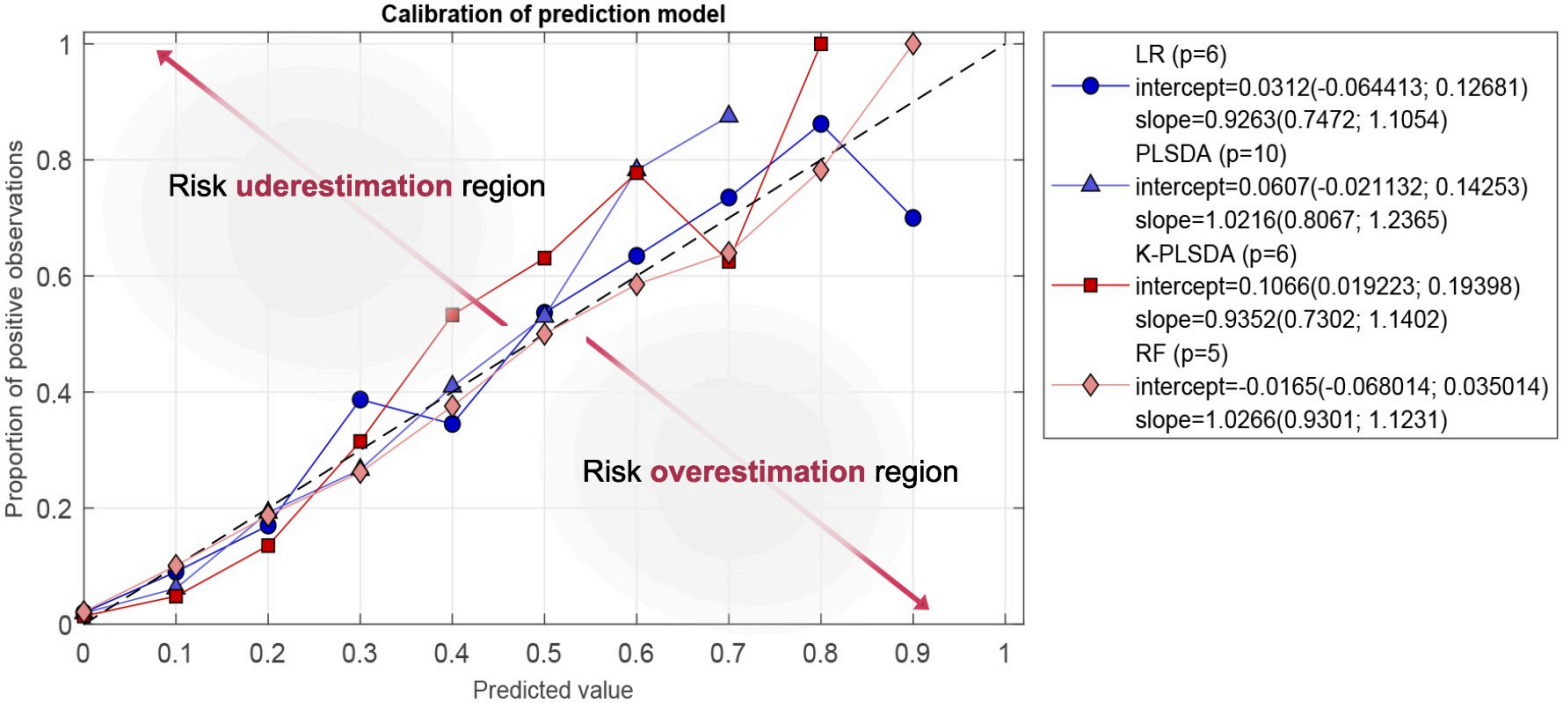
Optimal calibration risk prediction curves. Observed mortality (%) vs. predicted risk of mortality for all the classification algorithms under study at their respective optimal variable number setting. Predicted risk values were rounded to the first decimal digit, i.e., predicted value 0.1 refers to predictions between 0.05 and 0.15.

Fig 6 shows the calibrated risk prediction curve for each classification technique at its optimal variable number setting. These optimal calibration curves are the closest ones to the dashed diagonal line. Curves located in regions aside the diagonal would indicate an underestimation of the mortality risk (leading to under-treatment) or an overestimation (leading to over-treatment).

**Fig 7:**
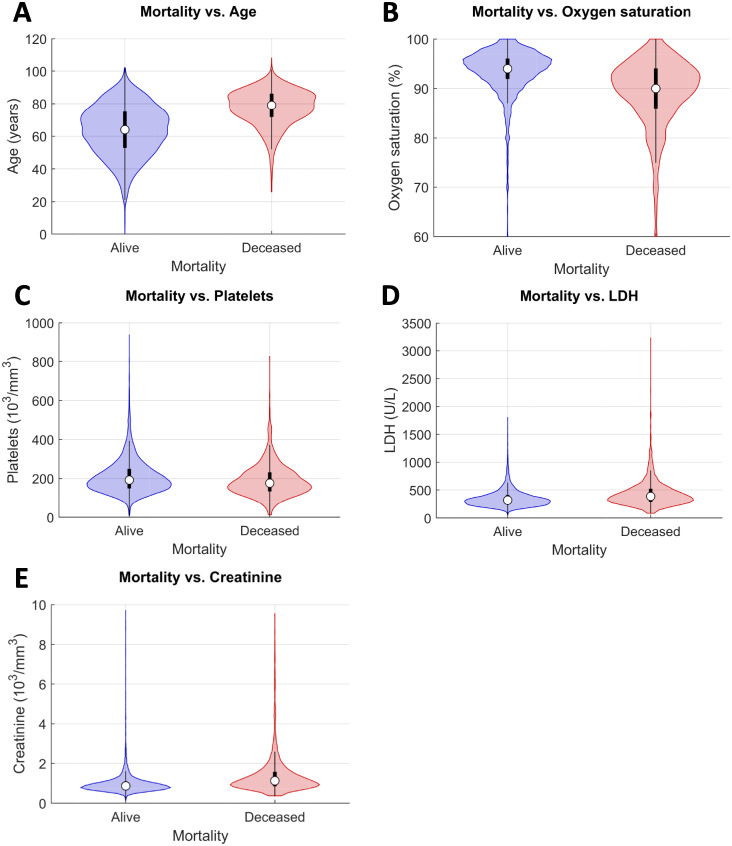
Marginal distributions of predictors used by the RF. Violin plots (blue: alive patients; red: deceased patients) for age (A), oxygen saturation (B), platelets (C), LDH (D), and creatinine (E).

In general, all the algorithms had a similar performance, although there were differences in terms of the optimal number of variables. Six variables (age, oxygen saturation, platelets, LDH, creatinine, and lymphocytes) were selected for LR and kernel PLSDA. The PLSDA model reached an optimal performance with ten predictors (from age to rf-24) and RF with five predictors (from age to creatinine). Consequently, to the light of the results, Random Forest was selected as the best classifier, showing slightly better results and with the minimum number of predictors. Fig 7 shows violin plots with the distribution of the five most important variables on this ranking for the deceased and the alive patients from the calibration data set.

The results yielded by the RF classification model suggested that five predictors encoded enough information to accurately predict the mortality risk of a given patient. These five predictors were then explored in the attempt of devising a simplified classifier based on them. The detailed procedure and the results obtained during intermediate steps of the mortality score confection can be found in the S1 File (pp 7–9).

**Fig 8:**
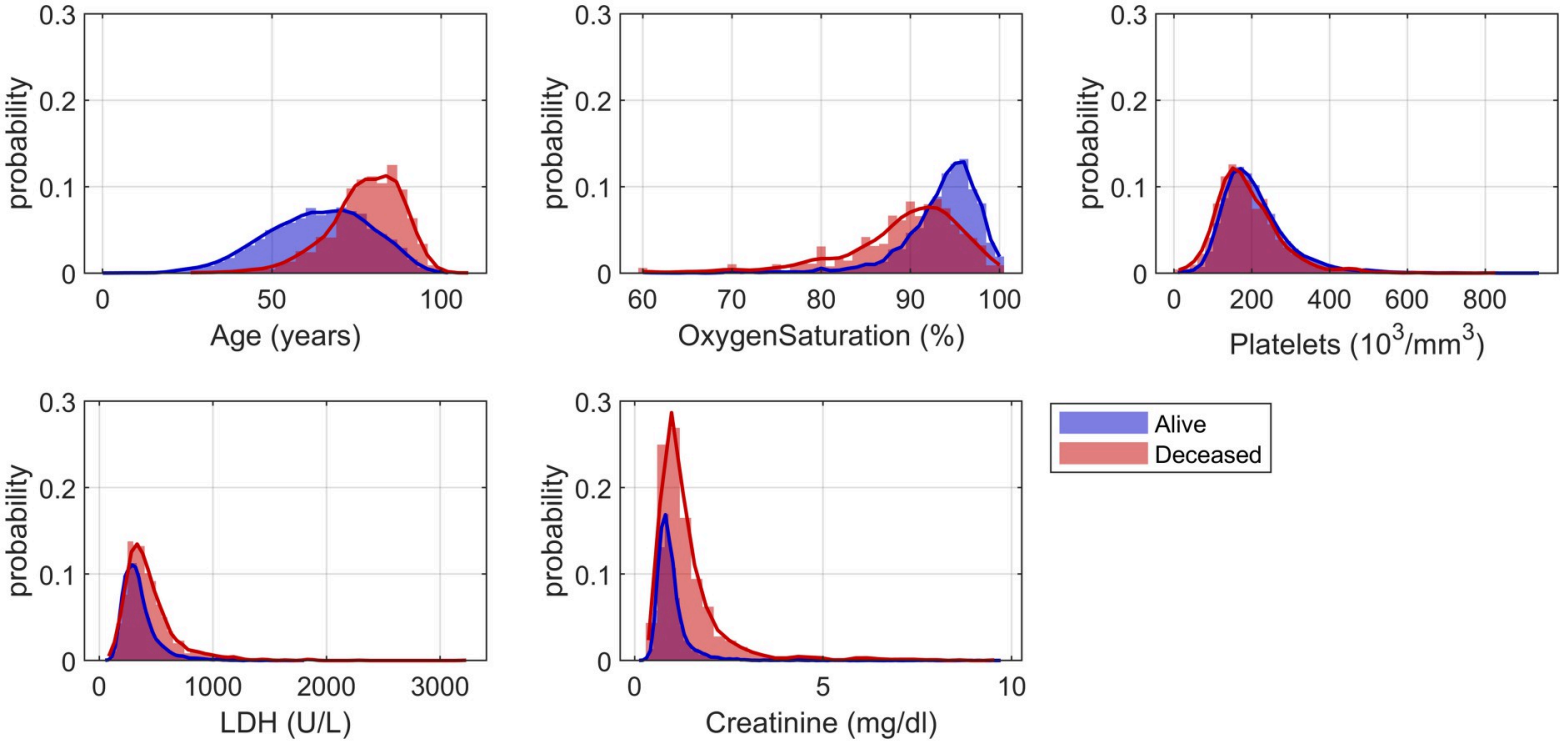
Histograms with marginal distributions of final set of predictors. Age, oxygen saturation, platelets, LDH and creatinine distribution within alive (blue) and deceased (red) patients.

Initially, the marginal distributions of age, oxygen saturation, platelets, LDH and creatinine for each class (“alive” and “deceased”), were inspected (Fig 8). Values of interest (such as the intersection points between the group distributions of each variable and the percentiles delimiting such distributions) were chosen as thresholds for each predictor.

**Fig 9:**
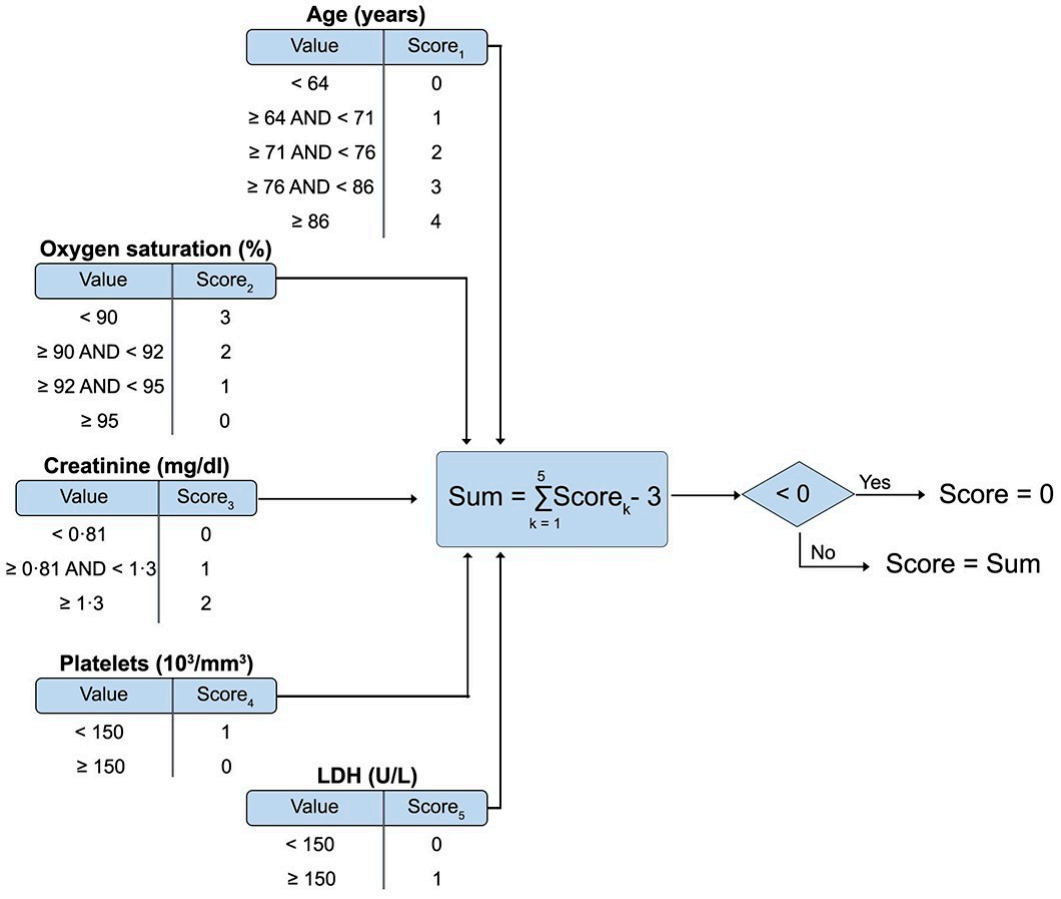
Final set of scoring rules. Formulation of the nine-levels mortality score for COVID-19 patients at their hospital admission.

**Fig 10:**
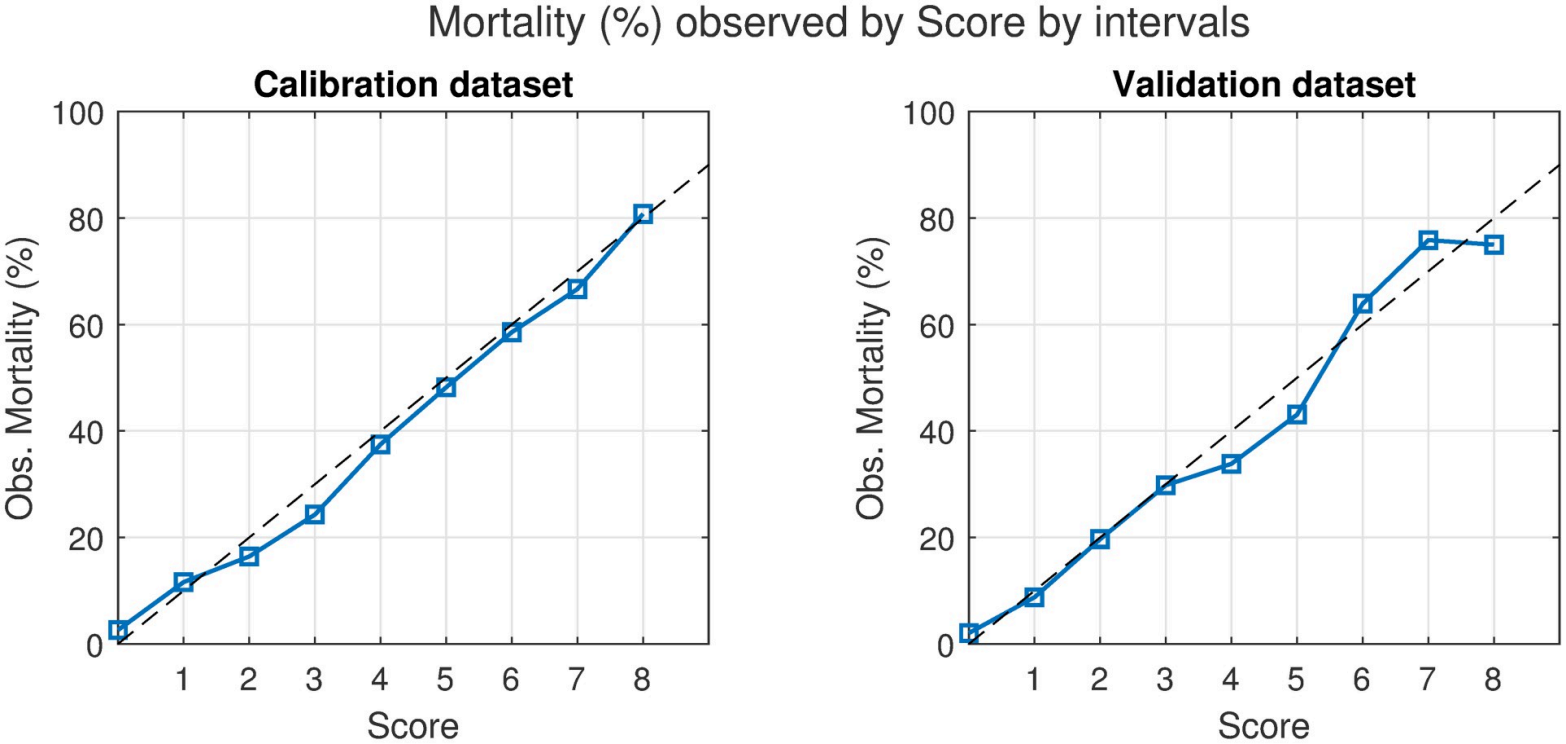
Observed mortality vs. score curves. Observed mortality at each level of the score for the Calibration data set and for the Validation data set.

Moreover, the importance of each variable according to the model was accounted as well to develop a realistic set of scoring rules (Fig 9). The final mortality score is a variable ranging from zero to eight, increasing with the risk of mortality as can be observed in Fig 10.

This information is displayed as well in the S1 File (p 9) and in S6 Table which shows the percentage of patients belonging to the two considered classes for each level of the mortality score.

## Discussion

In this work, we applied machine learning and multivariate statistical classification techniques to data prospectively collected from COVID-19 hospitalized patients in all regions of Spain to build a model for the prediction of their mortality risk at their hospitalization. The final model encompassed five predictors (age, oxygen saturation, creatinine, platelets, and LDH) and was trained based on the Random Forest algorithm. It returned in external validation (when patients not considered for model training and optimization were to be assessed) an AUC of 0.8454.

Virtually all published studies on COVID-19 populations agree that both age and oxygen saturation at hospital admission are closely related to the likelihood of death [4, 15–24]. Besides, COVID-19 mortality is strongly linked to an specific inflammation process and a coagulation disorder. There are patients who develop a severe inflammatory syndrome, which results in uncontrolled activation of the immune system and a massive release of pro-inflammatory cytokines, which translates into an increase in acute-phase reactants such as C-reactive protein, interleukin-6, ferritin, cell destruction markers such as LDH, and an increase in pro-inflammatory cells such as neutrophils [15, 17, 21, 22, 25, 26].

Another complication that results in high mortality in these patients is coagulation disorders. COVID-19 results in a systemic hypercoagulation state, producing pulmonary thromboembolisms, ischemic strokes, and other disorders, and a markedly large number patients experience severe complications. This complication can be assessed on the basis of 2 laboratory parameters: D-Dimer and platelets [16, 18]. Most prognostic studies also identified creatinine or urea as important factors related to mortality risk. [4, 15–24, 27]. Our data showed that the laboratory parameter that most influences mortality in relation to renal function is creatinine, which indicates whether renal filtering is effective.

Model calibration was carried out exploiting exclusively information that can be easily recorded at the admission to the hospital of COVID-19 patients. However, even if this information is available at the early stages of their hospitalization, we also tried to reduce as much as possible the number of variables to obtain an accurate mortality risk prediction without compromising its quality and performance.

A clear strength of our work is that the original database contains routinely obtained clinical data easily available at the hospital admission of this kind of patients. Age, oxygen saturation, platelets, LDH, and creatinine, have been previously identified among the core variables related to both morality and severe disease development after SARS-CoV-2 infection in multiple epidemiological studies. These five variables were also found to be the most predictive features in our study, independently on the classification algorithm utilized. In practical terms, this means that, for a given patient, just by recording the values for these five clinical parameters, predictive models could accurately estimate his/her mortality risk. All these features are coherent with the information already available about this disease [4, 22, 23]. Our results showed that the probability of mortality for a COVID-19 depends on variables of different nature, and not exclusively on those associated to respiratory functions. In addition to making models more parsimonious, reducing the number of predictors to a minimum set that is easy to measure also enables an easy implementation of this predictive strategy for its clinical use and for further validation with other data sets.

Another strength of this analytical approach lies in the sample size, a prospectively recruited cohort of 12,509 patients, including more than 2,000 deceased individuals. The sample size of previous studies on mortality among COVID-19 inpatients performed at Spanish hospitals ranged between 2,000 and 4,000 individuals, [18, 28] with 6.5% and 28.0% of mortality, respectively. Conversely, other studies with a larger sample size could not achieve proper predictive models [29]. Besides, the huge number of articles submitted during the pandemic in 2020, forced editorial offices (even of well-known biomedical journals) to change their policies due to scandals and polemics related to the reliability of the published data [30].

Furthermore, a systematic approach was implemented to compare statistical and machine learning algorithms not only in terms of classification performance but also in terms of model inference on the predictors. This is a very powerful (but barely used) approach that enables a more comprehensive assessment in terms of inferential coherence among different methodological strategies, which otherwise would be simply exploited as black-box techniques. Consequently, we consider that this additional validation increases the reliability of our results.

Our study showed, though, several limitations. This model was fitted in one of the worst moments of the pandemic. The patients included in this study were hospitalized during the first period of the pandemic, so the clinical characteristics of patients in our country today could be different [31]. Moreover, now, in 2022, patients could have different outcomes given the better knowledge of COVID-19 disease and the coverage of the vaccination campaign. However, in many countries—especially less developed ones—the situation may differ greatly from the scenario our current scenario here, and our findings may still be clinically useful there. In any case, our final prediction model should, indeed, be tested with an updated and more recent picture of the COVID-19 situation. Although it is not clear if the predictors of mortality have changed, new conditions may overall result in lower mortality rates also for patients with a high-risk profile. Such an assessment constitutes one of the main objectives of our future research work.

In conclusion, we used several statistical and machine learning approaches to obtain a data-driven model based on variables that could be easily acquired at COVID-19 patients’ admission to the hospital for the determination of their mortality risk. This resulted in a final model based on five predictors (age, oxygen saturation, platelets, LDH, and creatinine) that yielded a highly satisfactory classification performance (with an AUC of 0.8454). The interpretation of this model and the investigation of the relationships between these five predictors and the mortality risk contributed to the definition of a mortality score for COVID-19 patients at their admission that can be easily calculated and easily interpreted (it linearly increases along with the mortality risk). Once it will be validated with a prospective cohort representative of the latest COVID-19 management protocols, the mortality prediction model could be used as a powerful tool for the early recognition of the gravity and priority needs of SARS-Cov-2-infected hospitalized patients.

## Supporting information

S1 Fig Classification metrics (in calibration—n = 10,008; 38 predictors) for the four machine learning algorithms under study. Bars indicate the median value of each coefficient, and intervals indicate the 2.5% and 97.5% percentiles, Numerical values are in S5 Table.
(PDF)

S2 Fig Classification metrics (in validation) yielded by models encompassing sequentially higher numbers of important variables. Details on the incremental computational strategy adopted here are given in “Statistical analysis methodology” in the Section Methods. The term TNR stands for True Negative Rate, TPR for True Positive Rate, Acc. for Accuracy, MCC for Matthew’s Correlation Coefficient and AUC for Area Under the Curve. Mathematical expressions to compute each metric are provided in S4 Table.
(PDF)

S1 File Supplementary methods appendix. Contains technical details about different steps addressed in separate sections: 1) Missing data study; 2) Outlier assessment; 3) Preliminary univariate tests; 4) Database partition; 5) Missing data imputation; 6) Metrics for model comparison; 7) Mortality score calculation. It contains its own figures and references.
(PDF)

S1 Table Characteristics of patients in the complete observations data set. Summary of the univariate tests based on the odds ratio yielded by univariate logistic regression models built between every predictor and the mortality response. p-values in bold are < 10^−6^. For each numerical predictor, the mean, and the standard deviation (in parentheses) values are indicated. For each categorical predictor, the number, and the percentage (in parentheses) of cases are reported. All variables from Table 1 not appearing in Table 2 are included, with a summary of the univariate tests performed on the complete data set.
(PDF)

S2 Table Characteristics of patients in the calibration data set. Summary of the univariate tests based on the odds ratio yielded by univariate logistic regression models built between every predictor and the mortality response. p-values in bold are < 10^−6^. For each numerical predictor, the mean, and the standard deviation (in parentheses) values are indicated. For each categorical predictor, the number, and the percentage (in parentheses) of cases are reported. All variables from Table 2 are included, with a summary of the univariate tests performed on the calibration data set.
(PDF)

S3 Table Characteristics of patients in the validation data set. Summary of the univariate tests based on the odds ratio yielded by univariate logistic regression models built between every predictor and the mortality response. p-values in bold are < 10^−6^. For each numerical predictor, the mean, and the standard deviation (in parentheses) values are indicated. For each categorical predictor, the number, and the percentage (in parentheses) of cases are reported. All variables from Table 2 are included, with a summary of the univariate tests performed on the validation data set.
(PDF)

S4 Table Metrics used to evaluate the performance of each classification algorithm. The term TP stands for True Positives (TPR for True Positive Rate), TN for True Negatives (TNR for True Negative Rate), FP for False Positives, FN for False Negatives.
(PDF)

S5 Table Evaluation metrics from S4 Table for the calibration data set obtained over the 100 folds of training and testing with the calibration data set. The same values are illustrated in S1 Fig. The classifier LR refers to Logistic Regression, PLS-DA to Partial Least Squares—Discriminant Analysis, KPLSDA to Kernel PLS-DA and RF to Random Forest. The parameters correspond to the 2.5% percentile (P2.5), to the 50% percentile (Median) and to the 97.5% percentile (P97.5).
(PDF)

S6 Table Percentage of observed mortality at each level of the score for the calibration and validation data sets. (PDF)

## References

[1] WHO Coronavirus (COVID-19) Dashboard | WHO Coronavirus (COVID-19) Dashboard With Vaccination Data, https://covid19.who.int/,

[2] Burn E, Tebé C, Fernandez-Bertolin S, Aragon M, Recalde M, Roel E, et al. The natural history of symptomatic COVID-19 during the first wave in Catalonia. Nature communications. 2021;12(1):777. doi: 10.1038/s41467-021-21100-y 33536436 PMC7858639

[3] Gustine JN, Jones D. Immunopathology of Hyperinflammation in COVID-19. The American journal of pathology. 2021;191(1):4–17. doi: 10.1016/j.ajpath.2020.08.009 32919977 PMC7484812

[4] Knight SR, Ho A, Pius R, Buchan I, Carson G, Drake TM, et al. Risk stratification of patients admitted to hospital with covid-19 using the ISARIC WHO Clinical Characterisation Protocol: development and validation of the 4C Mortality Score. BMJ. 2020;370:22. doi: 10.1136/bmj.m3339 32907855 PMC7116472

[5] Wynants L, Van Calster B, Collins GS, Riley RD, Heinze G, Schuit E, et al. Prediction models for diagnosis and prognosis of covid-19: systematic review and critical appraisal. BMJ. 2020;369:m1328. doi: 10.1136/bmj.m1328 32265220 PMC7222643

[6] Van Dam PMEL, Zelis N, Van Kuijk SMJ, Linkens AEMJH, Brüggemann RAG, Spaetgens B, et al. Performance of prediction models for short-term outcome in COVID-19 patients in the emergency department: a retrospective study. Annals of medicine. 2021;53(1):402–409. doi: 10.1080/07853890.2021.1891453 33629918 PMC7919920

[7] Spanish Society of Hospital Pharmacy. Spanish Registry of treatment efficacy against SARS-CoV-2 COVID-19. Jerez de la Frontera: European Network of Centres for Pharmacoepidemiology and Pharmacovigilance; 2020. Available from: http://www.encepp.eu/encepp/viewResource.htm?id=34344.

[8] Harris PA, Taylor R, Minor BL, Elliott V, Fernandez M, O’neal L, et al. The REDCap consortium: Building an international community of software platform partners. Journal of biomedical informatics. 2019;95:103208. doi: 10.1016/j.jbi.2019.103208 31078660 PMC7254481

[9] McLachlan GJ. Discriminant Analysis and Statistical Pattern Recognition. Wiley Series in Probability and Statistics. Hoboken, NJ, USA: John Wiley & Sons, Inc.; 1992. Available from: http://doi.wiley.com/10.1002/0471725293.

[10] Barker M, Rayens W. Partial least squares for discrimination. Journal of Chemometrics. 2003;17(3):166–173. doi: 10.1002/cem.785

[11] Schölkopf B, Smola AJ, Bach F. Learning with kernels: support vector machines, regularization, optimization, and beyond. MIT press; 2001.

[12] Breiman L. Random forests. Machine Learning. 2001;45(1):5–32. doi: 10.1023/A:1010933404324

[13] Van Calster B, McLernon DJ, Van Smeden M, Wynants L, Steyerberg EW, Bossuyt P, et al. Calibration: The Achilles heel of predictive analytics. BMC Medicine. 2019;17(1):1–7. doi: 10.1186/s12916-019-1466-7 31842878 PMC6912996

[14] González-Cebrián A, Borràs-Ferris J, Ordovás-Baines JP, Hermenegildo—Caudevilla M, Climente—Martí M, et al. PROCOVID dataset, 2022. 10.5281/zenodo.6948496 PMC9499271 36137106

[15] Vaid A, Somani S, Russak AJ, De Freitas JK, Chaudhry FF, Paranjpe I, et al. Machine Learning to Predict Mortality and Critical Events in a Cohort of Patients With COVID-19 in New York City: Model Development and Validation. Journal of medical Internet research. 2020;22(11):e24018. doi: 10.2196/24018 33027032 PMC7652593

[16] Murri R, Lenkowicz J, Masciocchi C, Iacomini C, Fantoni M, Damiani A, et al. A machine-learning parsimonious multivariable predictive model of mortality risk in patients with Covid-19. Scientific Reports. 2021;11(1):21136. doi: 10.1038/s41598-021-99905-6 34707184 PMC8551240

[17] Domínguez-Olmedo JL, Gragera-Martínez Á, Mata J, Pachón Álvarez V. Machine Learning Applied to Clinical Laboratory Data in Spain for COVID-19 Outcome Prediction: Model Development and Validation. Journal of Medical Internet Research. 2021;23(4):e26211. doi: 10.2196/26211 33793407 PMC8048712

[18] Torres-Macho J, Ryan P, Valencia J, Pérez-Butragueño M, Jiménez E, Fontán-Vela M, et al. The PANDEMYC Score. An Easily Applicable and Interpretable Model for Predicting Mortality Associated With COVID-19; 2020. 10.3390/jcm9103066 PMC7598151 32977606

[19] Berry DA, Ip A, Lewis BE, Berry SM, Berry NS, MrKulic M, et al. Development and validation of a prognostic 40-day mortality risk model among hospitalized patients with COVID-19. PLOS ONE. 2021;16(7):e0255228. doi: 10.1371/journal.pone.0255228 34329317 PMC8323891

[20] Hajifathalian K, Sharaiha RZ, Kumar S, Krisko T, Skaf D, Ang B, et al. Development and external validation of a prediction risk model for short-term mortality among hospitalized U.S. COVID-19 patients: A proposal for the COVID-AID risk tool. PLOS ONE. 2020;15(9):e0239536. doi: 10.1371/journal.pone.0239536 32997700 PMC7526907

[21] Guan X, Zhang B, Fu M, Li M, Yuan X, Zhu Y, et al. Clinical and inflammatory features based machine learning model for fatal risk prediction of hospitalized COVID-19 patients: results from a retrospective cohort study. Annals of Medicine. 2021;53(1):257–266. doi: 10.1080/07853890.2020.1868564 33410720 PMC7799376

[22] Liang W, Liang H, Ou L, Chen B, Chen A, Li C, et al. Development and Validation of a Clinical Risk Score to Predict the Occurrence of Critical Illness in Hospitalized Patients With COVID-19. JAMA Internal Medicine. 2020;180(8):1081–1089. doi: 10.1001/jamainternmed.2020.2033 32396163 PMC7218676

[23] Yadaw AS, Li Yc, Bose S, Iyengar R, Bunyavanich S, Pandey G. Clinical features of COVID-19 mortality: development and validation of a clinical prediction model. The Lancet Digital Health. 2020;2(10):e516–e525. doi: 10.1016/S2589-7500(20)30217-X 32984797 PMC7508513

[24] Halasz G, Sperti M, Villani M, Michelucci U, Agostoni P, Biagi A, et al. A Machine Learning Approach for Mortality Prediction in COVID-19 Pneumonia: Development and Evaluation of the Piacenza Score. Journal of Medical Internet Research. 2021;23(5):e29058. doi: 10.2196/29058 33999838 PMC8168638

[25] Chubb H, Williams SE, Whitaker J, Harrison JL, Razavi R, Neill MO. Diagnostic Electrophysiology & Ablation Cardiac Electrophysiology Under MRI Guidance: an Emerging Technology Diagnostic Electrophysiology & Ablation. Arrhythmia & Electrophysiology Review. 2017;6(Ivc):85–93. doi: 10.15420/aer.2017 28845235 PMC5517375

[26] Henry BM, de Oliveira MHS, Benoit S, Plebani M, Lippi G. Hematologic, biochemical and immune biomarker abnormalities associated with severe illness and mortality in coronavirus disease 2019 (COVID-19): a meta-analysis. Clinical Chemistry and Laboratory Medicine (CCLM). 2020;58(7):1021–1028. doi: 10.1515/cclm-2020-0369 32286245

[27] Chen R, Liang W, Jiang M, Guan W, Zhan C, Wang T, et al. Risk Factors of Fatal Outcome in Hospitalized Subjects With Coronavirus Disease 2019 From a Nationwide Analysis in China. Chest. 2020;158(1):97–105. doi: 10.1016/j.chest.2020.04.010 32304772 PMC7158802

[28] Berenguer J, Ryan P, Rodríguez-Baño J, Jarrín I, Carratalà J, Pachón J, et al. Characteristics and predictors of death among 4035 consecutively hospitalized patients with COVID-19 in Spain. Clinical Microbiology and Infection. 2020;26(11):1525–1536. doi: 10.1016/j.cmi.2020.07.024 32758659 PMC7399713

[29] Casas-Rojo JM, Antón-Santos JM, Millán-Núñez-Cortés J, Lumbreras-Bermejo C, Ramos-Rincón JM, Roy-Vallejo E, et al. Clinical characteristics of patients hospitalized with COVID-19 in Spain: Results from the SEMI-COVID-19 Registry. Revista clínica española. 2020;220(8):480–494. doi: 10.1016/j.rce.2020.07.003 PMC7368900 33994573

[30] Group TEOTL. Learning from a retraction. The Lancet. 2020;396(10257):1056. doi: 10.1016/S0140-6736(20)31958-9 PMC7498225 32950071

[31] Castro-Balado A, Varela-Rey I, Bandín-Vilar EJ, Busto-Iglesias M, García-Quintanilla L, Mondelo-García C, et al. Clinical research in hospital pharmacy during the fight against COVID-19. Farmacia hospitalaria. 2020;44(7):66–70. 32533675 10.7399/fh.11494

