# Supplementary material for: Machine-learning-derived predictive score for early estimation of COVID-19 mortality risk in hospitalized patients
Source: PLoS One. 2022 Sep 22;17(9):e0274171. doi: 10.1371/journal.pone.0274171 (PMC9499271; doi:10.1371/journal.pone.0274171)
Supplement: S1 Fig — Bars indicate the median value of each coefficient, and intervals indicate the 2.5% and 97.5% percentiles, Numerical values are in S5 Table. (PDF) [file pone.0274171.s001.pdf]

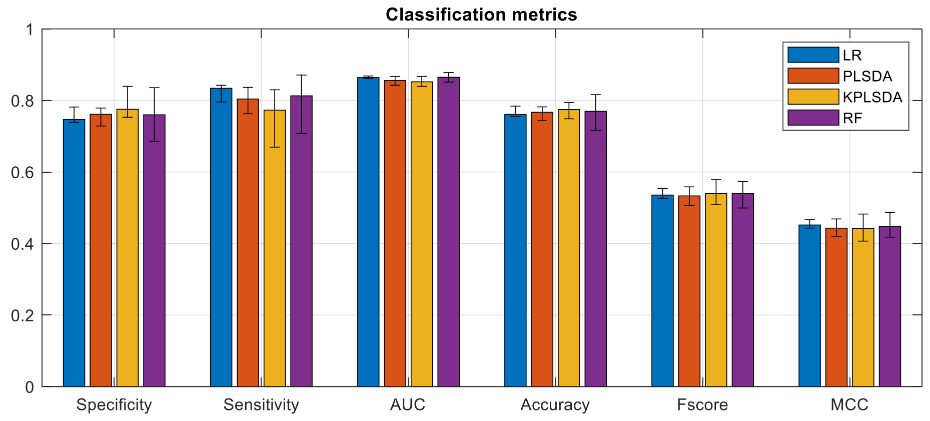

S1 Figure. Classification metrics (in calibration – n = 10008; 38 predictors) for the four machine learning algorithms under study. Bars indicate the median value of each coefficient, and intervals indicate the 2.5% and 97.5% percentiles. Numerical values are in S5 Table**.**
