# Supplementary material for: Machine-learning-derived predictive score for early estimation of COVID-19 mortality risk in hospitalized patients
Source: PLoS One. 2022 Sep 22;17(9):e0274171. doi: 10.1371/journal.pone.0274171 (PMC9499271; doi:10.1371/journal.pone.0274171)
Supplement: S2 Fig — Details on the incremental computational strategy adopted here are given in “Statistical analysis methodology” in the Section Methods. The term TNR stands for True Negative Rate, TPR for True Positive Rate, Acc. for Accuracy, MCC for Matthew’s Correlation Coefficient and AUC for Area Under the Curve. Mathematical expressions to compute each metric are provided in S4 Table. (PDF) [file pone.0274171.s002.pdf]

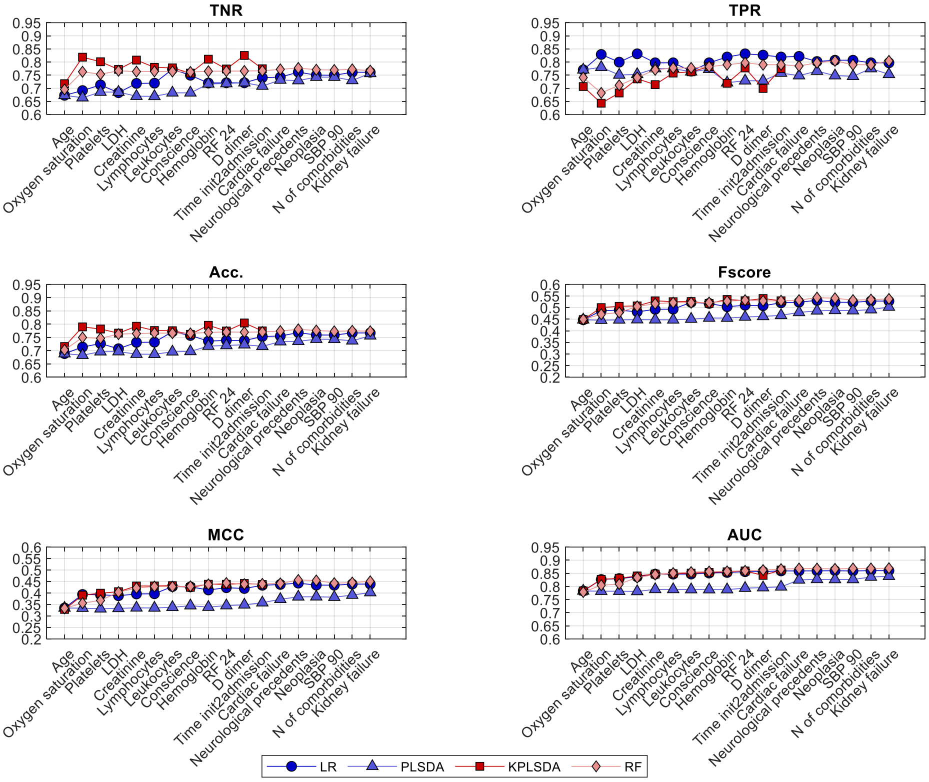

S2 Figure. Classification metrics (in validation) yielded by models encompassing sequentially higher numbers of important variables. Details on the incremental computational strategy adopted here are given in “Statistical analysis methodology” in the Section Methods, from the TNR (True Negative Rate), TPR (True Positive Rate), Acc, (Accuracy), MCC for Matthew’s Correlation Coefficient and AUC for Area Under the Curve. Mathematical expressions to compute each metric are provided in S4 Table.
