# Supplementary material for: Machine-learning-derived predictive score for early estimation of COVID-19 mortality risk in hospitalized patients
Source: PLoS One. 2022 Sep 22;17(9):e0274171. doi: 10.1371/journal.pone.0274171 (PMC9499271; doi:10.1371/journal.pone.0274171)
Supplement: S1 File — Contains technical details about different steps addressed in separate sections: 1) Missing data study; 2) Outlier assessment; 3) Preliminary univariate tests; 4) Database partition; 5) Missing data imputation; 6) Metrics for model comparison; 7) Mortality score calculation. It contains its own figures and references. (PDF) [file pone.0274171.s003.pdf]

## Appendix for "Machine-learning-derived predictive score for early estimation of COVID-19 mortality risk in hospitalized patients"

### Supplementary Methods

### Database "cleaning": Missing data study

This section summarizes the process of "cleaning" and imputing the missing values within the original database of the study until reaching a final basal dataset with complete observations. This goal required finding a balance between the number of variables and observations that would be removed, trying to minimize the loss of information, and, at the same time, the impact that the imputation could have on the interpretation of the data modeling results.

Given the unbalancedness between alive and deceased individuals (81,79% vs. 18,21%), our priority was to preserve as many patients of the latter class as possible since they constitute the limiting category. Moreover, ICU-related variables were not considered as not all patients underwent ICU treatment or monitoring, which induced the presence of missing values not directly imputable by standard approaches typically utilized for this purpose.

Similarly, nested variables (e.g., the dose of drug A is nested to the fact that a patient has received the corresponding treatment or not) were also removed**.** Other variables, such as the Body Mass Index (BMI), Lymphocytes to C-reactive protein Ratio (LCR), Platelets to Lymphocytes Ratio (PLR), and Neutrophils to Lymphocytes Ratio (NLR), were included since they could be potential biomarkers for mortality.

Considering all these aspects, the initial database was constituted of 15,628 rows (2,846 deceased and 12,782 alive patients) and 48 variables (Supplementary Methods Figure 1).

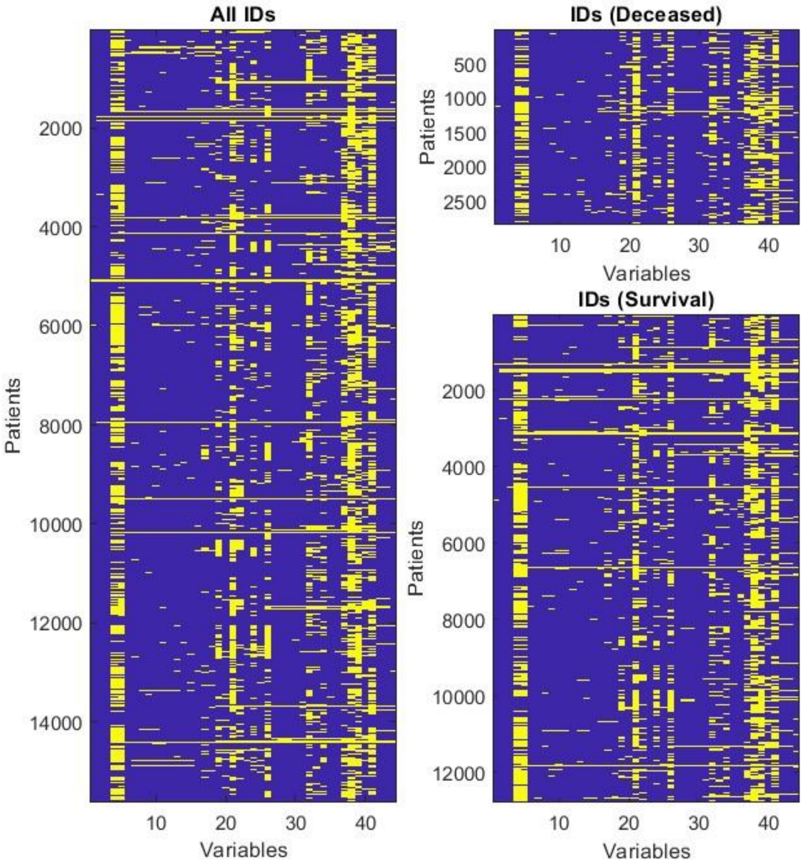


**Supplementary Methods Figure 1:** Representation of the missing (yellow) and complete (blue) data records within the dataset containing all the observations (left), in the subset containing only

observations related to deceased patients (upper right), and in the subset containing only observations related to alive patients (lower right).

Investigating the distribution of missing values across patients (*i.e.*, database rows), it was observed that for the group of alive patients, approximately 757 (6%) out of the 12,782 individuals showed more than 30% of missing data records. This percentage increases up to 8.4% (120/2,846) for the deceased class (Supplementary Methods Figure 2).

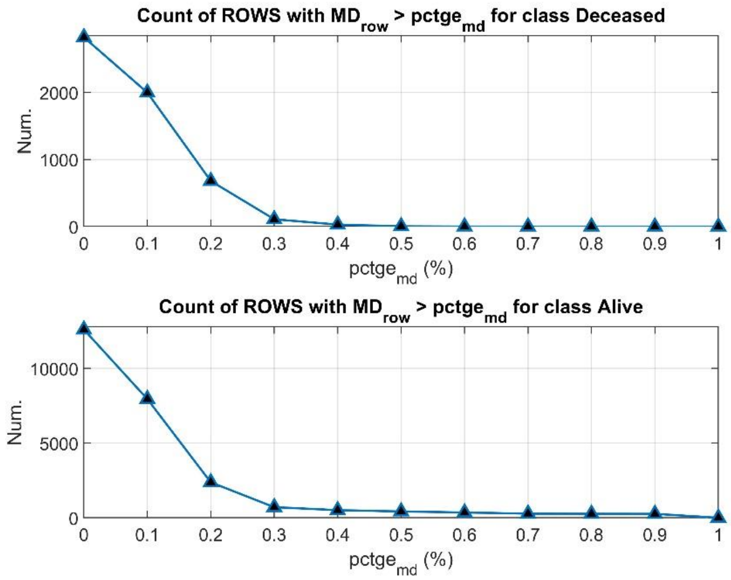


**Supplementary Methods Figure 2:** Number of patients with a percentage of missing values beyond the values expressed along the x-axis for the deceased (up) and the alive group of patients (down).

Thus, a first cut-off point was established to filter out observations with a percentage of missing entries larger than 30% for the alive patients. Supplementary Methods Figure 3 shows the missing data pattern related to these observations. It seems to be very similar for most of them, which means that the same type of information was missing/not recorded for a major part of these individuals.

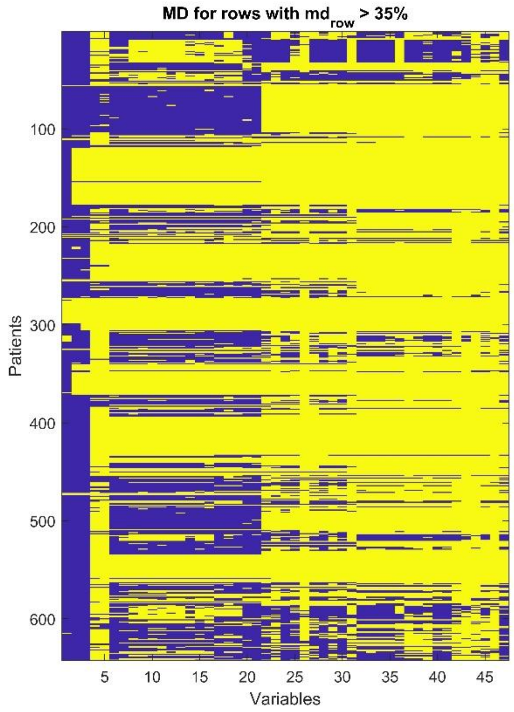


**Supplementary Methods Figure 3:** Representation of the missing (yellow) and complete (blue) data records within the 1,265 rows preliminarily excluded from the initial dataset.

After this first step, the residual percentage of missing values was assessed variable-wise (*i.e.*, database columns). As one can easily deduce from Supplementary Methods Table 1, the number of variables with over 50% of missing records, was practically the same for both classes of patients. Those exhibiting more than 50% of missing entries for the alive individuals were, therefore, removed.

**Supplementary Methods Table 1:** Percentage of missing values for the variables measured in this study (sorted in descending order). Only those with over 35% of missing records are listed for each category of patients under study.

| **Deceased patients** | | **Alive patients** | |
|---|---|---|---|
| **Missing Data (%)** | **Variable Name** | **Missing Data (%)** | **Variable Name** |
| 65,57% | height | 63,17% | lactic acid |
| 62,27% | weight | 53,29% | height |
| 52,26% | glasgow | 52,89% | glasgow |
| 51,58% | lactic acid | 50,48% | ferritin |
| 51,36% | creatine kinase | 48,35% | weight |
| 49,28% | ferritin | 47,98% | creatine kinase |
| 41,32% | affected quadrants | 35,38% | procalcitonin |
| 26,90% | procalcitonin | 33,37% | affected quadrants |

This alternating "cleaning" procedure was iterated until variables such as "affected quadrants", "curb65" or "oxygen saturation", appeared as the next ones to be deleted because of their missing data percentage (Supplementary Methods Table 2). Given their medical relevance, they needed to be kept in the final dataset. For this reason, we decided to stop this row/column selection at this point, which yielded a database containing 10,515 alive and 2085 deceased individuals, each one with less than 20% of missing data records (Supplementary Methods Figure 4).

**Supplementary Methods Table 2:** Percentage of missing values for the variables measured in this study (sorted in descending order). Only those with over 10% of missing records are listed for each category of patients under study.

| Deceased patients | | Alive patients | |
|---|---|---|---|
| Missing Data (%) | Variable Name | Missing Data (%) | Variable Name |
| 31,77% | affected quadrants | 31,70% | affected quadrants |
| 31,29% | procalcitonin | 29,05% | procalcitonin |
| 29,08% | aspartate transaminase | 26,80% | aspartate transaminase |
| 16,99% | d-dimer | 14,75% | respiratory frequency < 24 bpm |
| 14,54% | lactate dehydrogenase | 14,67% | oxygen saturation |
| 13,82% | oxygen saturation | 12,56% | lactate dehydrogenase |
| 13,53% | respiratory frequency < 24 bpm | 10,21% | d-dimer |
| 7,20% | alanine transaminase | 6,63% | alanine transaminase |

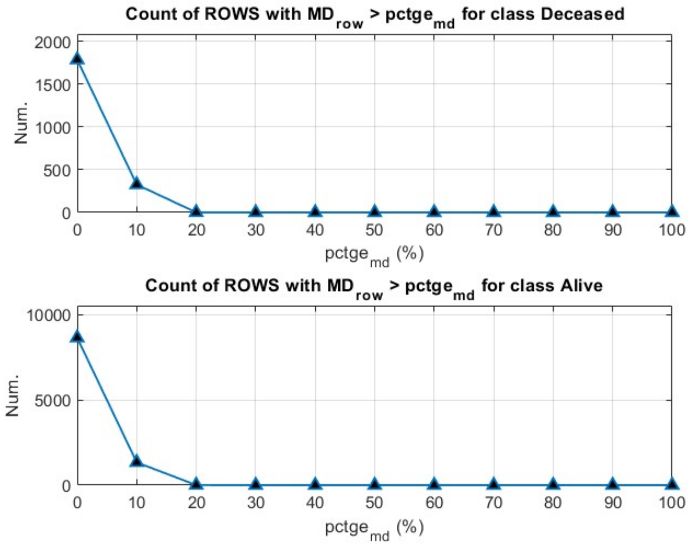


**Supplementary Methods Figure 4:** Number of patients with a percentage of missing values beyond the values expressed along the x-axis for the deceased group (up) and the alive group (down).

This database was then used to obtain one with complete patient observations (*i.e.*, without missing data), consisting of 36 variables, 158 deceased and 1,243 alive individuals. If some of the previously removed variables were found to have complete records for this subset of patients, they were finally re-integrated into the ultimate data structure.

Supplementary Methods Figure 5 shows a bar plot with the percentage of missing data for the remaining removed variables within the patients gathered in the complete database. As can be seen, most of them show more than 25% of missing entries. If complete observations were to be kept, considering these variables would, thus, imply reducing, even more, the size of the sample under study. Considering that an already substantial reduction of the number of observations was performed (only data for 9.72% of alive patients and 5,55% of deceased patients were finally analyzed), it was decided not to re-include any of them.

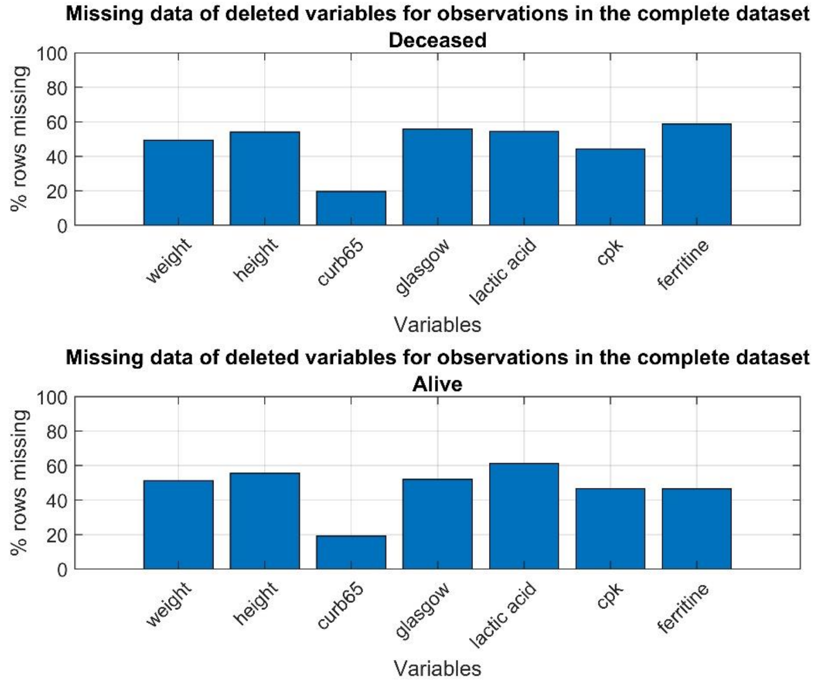


**Supplementary Methods Figure 5:** Percentage of missing entries within the measured variables excluded by the "cleaning" procedure (appendix p. 1-3) for the deceased (above) and the alive (below) patients included in the complete database.

**Database "cleaning": Outlier assessment**

In a second step, the complete database was studied by Principal Component Analysis (PCA) [1]. PCA is a multivariate statistical tool that enables its exploratory analysis and the detection of potential outlying observations. Outlying observations should not be considered as part of the same population represented by the rest of the dataset (for them, in fact, certain variables might show abnormal values due to, *e.g.*, measurement errors or inconsistency in the units). Mathematically, outlier removal is justified given the distortions that the corresponding observations can induce in the estimated parameter of the fitted model and that can bias the conclusions of the study. Detecting anomalous observations and studying the reasons behind their anomalous behavior is key before any further use of a particular dataset.

Here, the Distance to the Model (DModX) of each individual was assessed to identify the presence of anomalous observations. The DModX statistic assumes abnormally high values when atypical patterns in the correlation structure of the measured variables are observed. As shown in Supplementary Methods Figure 6, only one patient (highlighted by a red circle) was found to be characterized by a relatively large DModX value.

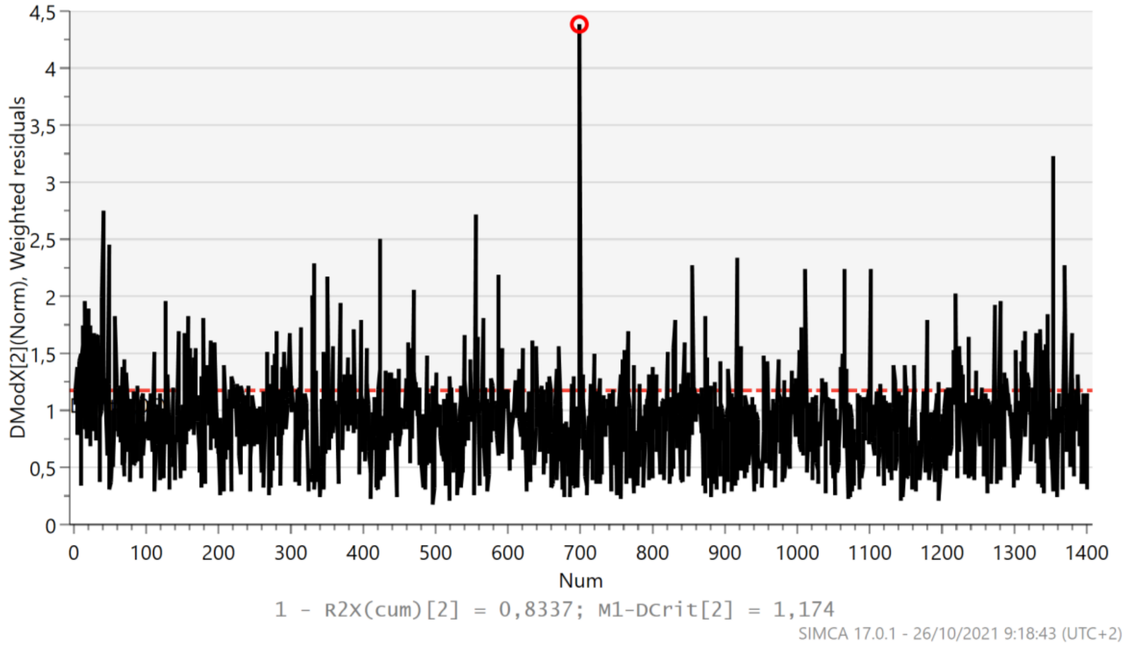


**Supplementary Methods Figure 6:** DModX (metric of orthogonal distance to the PCA model) values (resulting from a 4-component PCA model) for the observations of the complete dataset.

When the contributions of each predictor to the DModX associated with the outlying patient are inspected, the highest one relates to NLR. Plotting the raw values of this variable for all the patients of the complete dataset highlights that the one for the concerned individual is relatively larger compared to those measured for all the other subjects (Supplementary Methods Figure 7).

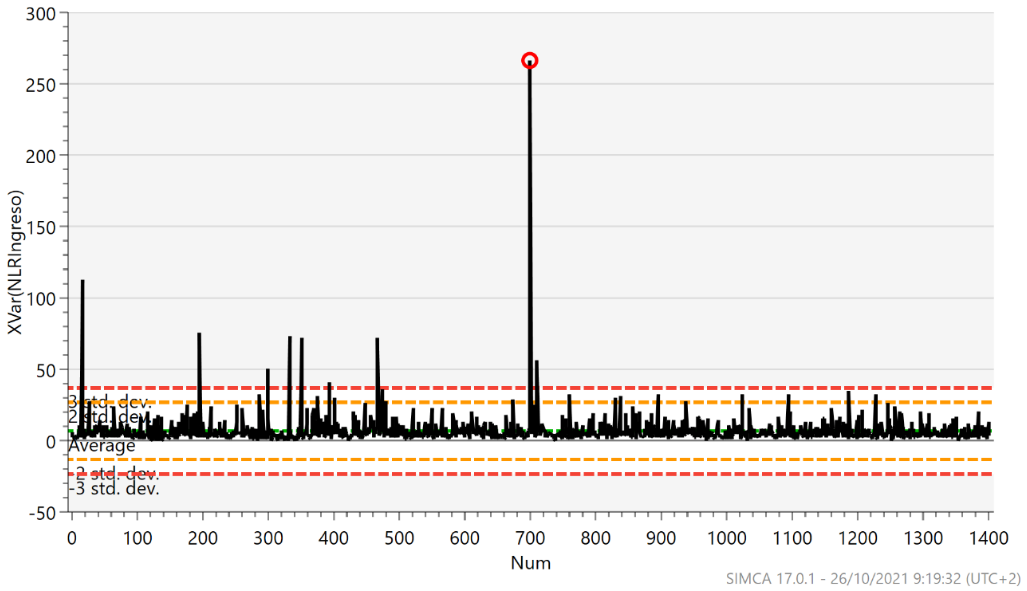


**Supplementary Methods Figure 7:** Raw values of the NLR variable for all the patients of the complete dataset. The outlying observation is highlighted by a red circle.

After the deletion of this observation, another PCA model was fitted, and no additional outliers were detected. Thus, the final database contains 1400 observations (11,21% of the mortality observed) and 38 predictors.

**Preliminary univariate tests**

Before obtaining more complex models, a preliminary univariate test was carried out on the complete observations of the Basal dataset (n = 1400). Since the response variable is categorical (0 for alive and 1 for deceased individuals), these tests were based on the Student's t-testing for numerical predictors and the $\chi^2$-testing for categorical predictors. Moreover, odd-ratios (ORs) were calculated for all predictors using a logistic regression model. The resulting *p*-values were stored. A threshold of 0·05 was exploited

to determine statistically significant coefficients. The results of this analysis can be found in Supplementary Table 2.

**Database partition**

The Basal dataset was randomly split (without replacement based on a uniform distribution) into two groups of independent patients: the former (comprising 80% of the sample) was used for model calibration (Calibration dataset, n=10,008), while the latter (including 20% of the sample) for model validation (Validation dataset, n=2,501) (Figure 1). It is important to highlight that in all consecutive partitions of the initial dataset, the proportion between deceased and alive patients (16.46% of mortality) was preserved, ensuring the consistency of the mortality ratio in both the calibration and validation datasets.

An iterative framework was designed, with 100 repetitions of model training and testing, enabling replacement across the 100 folds (Figure 1). This lets us account for the potential variability that one would expect due to the fact of using a slightly different dataset for model fitting and testing, which can become useful to handle future datasets. Each fold contained a partition of the calibration dataset into a training subset with 75% of the observations from the Calibration dataset and a testing dataset including the remaining 25% of the individuals. The mortality percentage of the original dataset was kept constant in each one of the partitions.

**Missing data imputation**

The technique used here for missing data imputation is an extension of Trimmed Scores Regression (TSR) capable of coping with categorical and integer variables. [2] This algorithm relies on the multivariate correlation among the observed variables, which is a better option for the imputation, compared to widespread univariate approaches.

**Calculation of variables' importance coefficients**

This step was necessary to identify the most important variables linked to COVID-19 mortality recorded at hospital admission. However, these coefficients were calculated differently for each classification algorithm, and they were an informative measure of both the importance of the predictor (in terms of magnitude) and the interpretation of this relation: positive coefficients denote risk factors and negative coefficients connote protection factors against the mortality by COVID-19.

For the **Logistic Regression** and **Partial Least Squares – Discriminant Analysis** models, the importance coefficient for each predictor was calculated as the median of the predictor coefficients on the models obtained over the 100 folds. If the predictor's coefficient was not statistically significant on the LR model (coefficient's p-value > 0.05), then it was equivalent to say that its value was statistically equivalent to zero. Thus, it was neither positive nor negative, and its sign was not considered to calculate the sign coherence over the 100 folds.

For the **kernel Partial Least Squares – Discriminant Analysis**, the model does not offer directly a coefficient reporting the relationship between the predictors and the response. However, in this work, we used an approach to estimate this information based on pseudosamples.[3]

Pseudosamples are fictitious samples built by matrices where all predictors except one remain constant on their average values. For the predictor that does not remain constant, a linear spacing between its minimum and maximum values is generated. The projection of this matrix on the classifier leads to a certain trajectory of the response, which can be correlated to the changes on the pseudosample matrix synthetically generated for that predictor.

Doing this procedure for each predictor yields the obtention of the trajectory for each predictor pseudosample matrix. The changes in the response trajectory can be parameterized to quantify in a single metric the effect of each predictor on the model's outcome. In the case of this work, the slopes of the response trajectory obtained with each pseudosamples matrix were calculated to measure the importance of each predictor. Then, the median coefficients were obtained for all predictors using the slopes calculated over the 100 folds.

In this case, the lack of a direct metric reporting the statistical significance of the predictors did not allow the possibility of having folds in which there were importance coefficients that could be

considered null in practical terms. Moreover, the outcome yielded by the kPLSDA model was initially continuous and then discretized by a threshold. Consequently, for the kPLSDA model, variable importance metrics were not null overall folds, having always a certain sign reporting the relationship between the variation in the response and the variation in the pseudosamples.

Finally, for the **Random Forest** model, the pseudosampling approach was also obtained. This was decided because, despite the existence of variable importance metrics for RF models, these metrics are not informative about the sign of the relationship between the predictor and the response. Hence, the pseudosampling approach was also applied for the RF model**,** as for the kPLSDA model.

Nonetheless, the RF model is an ensemble of Classification Trees, which meant that the output was always, by definition, discrete. Thus, there were some cases in which there was not any variation in the classification associated to the variation of the pseudosample, i.e., the label assigned was always the same for all pseudosamaples obtained for a given predictor. In these cases, the variable importance metric, computed as the quotient between the variation in the response and the variation in the pseudosample, had a zero value for the variation in the response term. This resulted in importance metrics equal to zero for some predictors in some folds. Similarly, as for LR and PLSDA importance metrics, the sign of these coefficients was not considered, yielding partially empty bars for some predictors in Figure 3D.

**Mortality score calculation**

As explained in the main text, the mortality predictive model must be implemented in a hospital environment, overcoming daily limitations that can prevent its use. For instance, reducing the number of necessary inputs for the score calculation can ease its implementation. Besides, the mortality score computation should be, in principle, device- or app-free, minimizing, this way, the required resources for its determination and interpretation. All this should be accounted for along with the main purpose, which is, in the end, to obtain an estimate of the mortality risk for a given patient at his/her hospitalization.

With this goal in mind, the set of five predictors selected for training the Random Forest classifier was used to define a COVID-19 mortality score. First, the marginal distributions of these predictors for each class of patients (alive and deceased) were inspected. Supplementary Methods Figure 8 shows these marginal distributions and the selection of the first cut-off points used for an initial dichotomization of the five predictors.

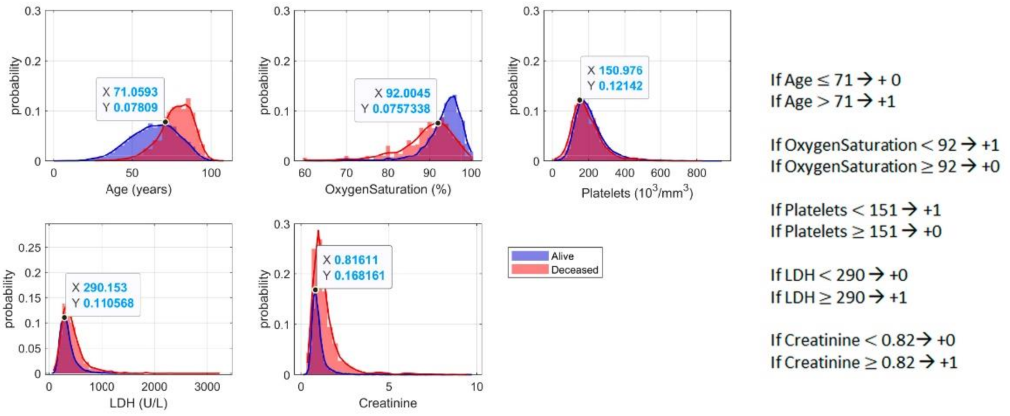


**Supplementary Methods Figure 8:** Histograms with kernel estimates of the corresponding probability functions for the five predictors under study. Blue and red bars/lines relate to alive and deceased patients, respectively. The cross-points of every pair of group distributions are high

Next, a regression model was fitted between these five dichotomized variables (as shown in Supplementary Methods Figure 8) and the mortality risk predicted by the RF model. The importance of

each variable in the mortality score definition can be, therefore, quantified as its respective regression coefficient. Supplementary Methods Figure 9 shows the results of this regression model.

```
calib_score_rules =
Linear regression model:
    risk_pred ~ 1 + Age + OxygenSaturation + Platelets + LDH + Creatinine

Estimated Coefficients:
                           Estimate        SE         tStat       pValue  
                          _________    _________    _______    __________

    (Intercept)           -0.048427    0.0035354    -13.698    2.5247e-42
    Age_1                   0.19263    0.0033408     57.659             0
    OxygenSaturation_1       0.1692    0.0037195     45.489             0
    Platelets_1            0.052905    0.0036726     14.405    1.3977e-46
    LDH_1                  0.046914     0.003407      13.77     9.447e-43
    Creatinine_1           0.064955    0.0033957     19.129    3.9037e-80


Number of observations: 10008, Error degrees of freedom: 10002
Root Mean Squared Error: 0.16
R-squared: 0.469,  Adjusted R-Squared: 0.469
F-statistic vs. constant model: 1.77e+03, p-value = 0
```

**Supplementary Methods Figure 9:** Results of the regression performed between the mortality risk predicted by the RF model and the five dichotomized predictors.

These coefficients were scaled by the minimum one and, afterward, rounded to the closest integer, which yielded a scale of variable relative importance for the mortality prediction (Supplementary Methods Table 3).

**Supplementary Methods Table 3:** Relative importance of the five dichotomized variables.

| Variable | Relative Importance | Round (Relative Importance) |
| --- | --- | --- |
| Age_1 | 3.60 | 4 |
| OxygenSaturation_1 | 2.60 | 3 |
| Platelets_1 | 1 | 1 |
| LDH_1 | 1.20 | 1 |
| Creatinine_1 | 1.60 | 2 |

The relative importance from Supplementary Methods Table 3 was used to establish more intervals for variables with relative importance above one. These new intervals were based on a search of characteristic points of the distributions, such as points of slope increase or decrease. The final scoring implementation is outlined in Figure 9. At first, applying these rules lead to a score ranging from zero to 11. However, since the first three levels of the score were grouping very little information about the mortality in the deceased group (Supplementary Methods Figure 10), they merged into the “zero” category, resulting in a final score with nine levels, ranging from zero to eight.

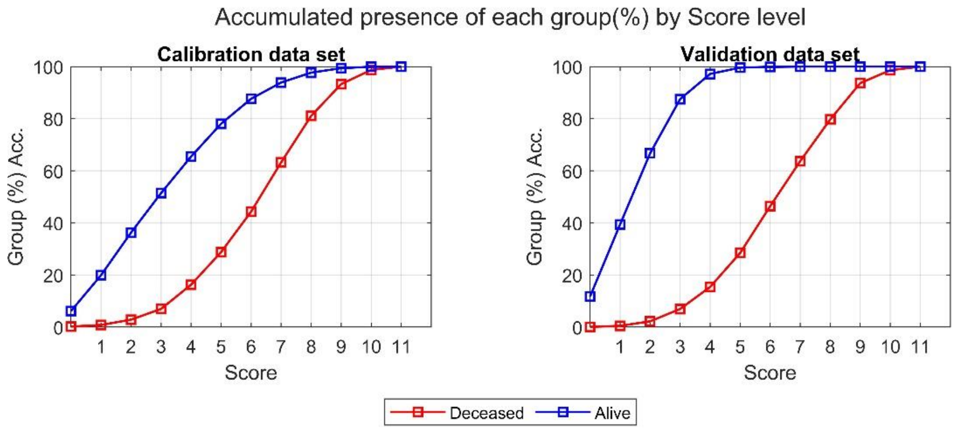


**Supplementary Methods Figure 10:** Accumulated distributions of deceased (red) and alive (blue) patients along the score values for the calibration dataset (left) and the validation dataset (right).

Finally, Supplementary Methods Figure 11 and S6 Table, show the marginal distributions (in absolute and percentage counts) of the patients of each considered class within every score interval.

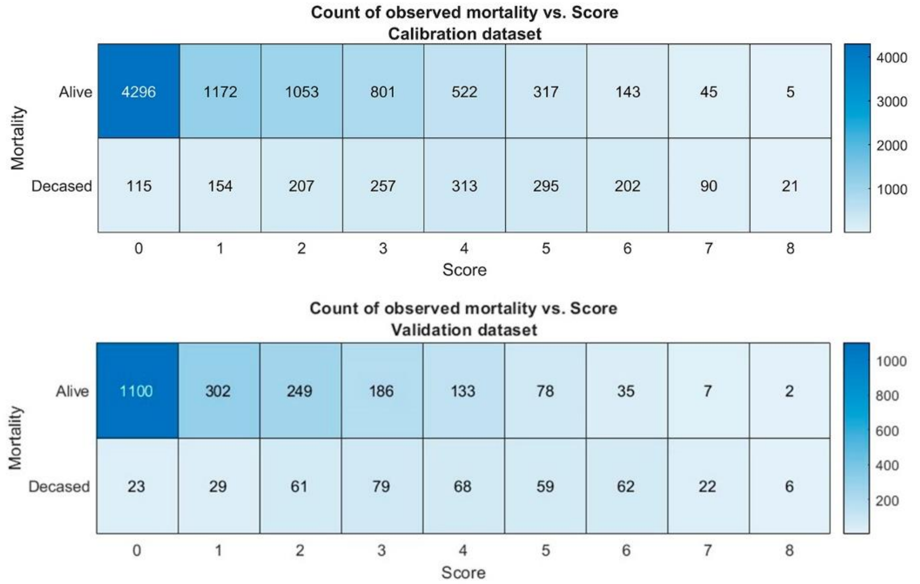


**Supplementary Methods Figure 11:** Contingency tables containing the number of individuals at each mortality score level for both classes of patients in the calibration (above) and validation (below) datasets.

## References


1 Wold S, Esbensen K, Geladi P. Principal component analysis. *Chemom Intell Lab Syst* 1987; **2**: 37–52.

2 Folch-Fortuny A, Arteaga F, Ferrer A. PCA model building with missing data: New proposals and a comparative study. *Chemom Intell Lab Syst* 2015; **146**: 77–88.

3 Postma GJJ, Krooshof PWTWT, Buydens LMCMC. Opening the kernel of kernel partial least squares and support vector machines. *Anal Chim Acta* 2011; **705**: 123–34.

4 Van Calster B, McLernon DJ, Van Smeden M, et al. Calibration: The Achilles heel of predictive analytics. *BMC Med* 2019; **17**: 1–7.
