# Supplementary material for: Machine-learning-derived predictive score for early estimation of COVID-19 mortality risk in hospitalized patients
Source: PLoS One. 2022 Sep 22;17(9):e0274171. doi: 10.1371/journal.pone.0274171 (PMC9499271; doi:10.1371/journal.pone.0274171)
Supplement: S1 Table — Summary of the univariate tests based on the odds ratio yielded by univariate logistic regression models built between every predictor and the mortality response. p-values in bold are < 10−6. For each numerical predictor, the mean, and the standard deviation (in parentheses) values are indicated. For each categorical predictor, the number, and the percentage (in parentheses) of cases are reported. All variables from Table 1 not appearing in Table 2 are included, with a summary of the univariate tests performed on the complete data set. (PDF) [file pone.0274171.s004.pdf]

S1 Table. Characteristics of patients in the Complete observations data set.

	Total; n = 1,400	Alive; n = 1,243	Deceased; n = 157	Odds ratio	p value
**Variable**	**m(s.d.) or n (%)**	**m(s.d.) or n (%)**	**m(s.d.) or n (%)**	**(95% CI)**	
Affected quadrants	2.16 (1)	2.12 (0.99)	2.5 (1.08)	1.43 (1.27;1.58)	<0.0001
Time init – urg, days	7.14 (4.83)	7.27 (4.64)	6.08 (6.07)	0.94 (0.89;0.98)	0.0028
Time init – micro, days	7.87 (6.33)	8.05 (6.42)	6.39 (5.35)	0.94 (0.9;0.98)	0.0012
Neutrophils, $10^3/mm^3$	6.48 (8.6)	6.35 (8.72)	7.57 (7.54)	1.01 (1;1.03)	0.01
Platelets, $10^3/mm^3$	208.63 (87.55)	210.14 (88)	196.66 (83.21)	1 (1;1)	0.07
CRP	88.6 (99.29)	84.27 (96.24)	122.92 (115.52)	1 (1;1)	<0.0001
PCT	0.58 (4.53)	0.58 (4.79)	0.59 (1.29)	1 (0.96;1.04)	0.98
N of comorbidities	1.22 (1.23)	1.13 (1.2)	1.92 (1.31)	1.57 (1.44;1.69)	<0.0001
NLR	6.55 (7.27)	6.06 (6.12)	10.4 (12.63)	1.06 (1.04;1.08)	<0.0001
LCR	0.28 (1.65)	0.3 (1.73)	0.12 (0.77)	0.8 (0.45;1.15)	0.21
PLR	231.51 (174.88)	225.7 (166.32)	277.56 (227.24)	1 (1;1)	0.0009
Sex				0.7 (0.35;1.05)	0.043
Man	822 (58.71%)	718 (57.76%)	104 (66.24%)		
Woman	578 (41.29%)	525 (42.24%)	53 (33.76%)		
HBP				2.64 (2.29;3)	<0.0001
No	737 (52.64%)	687 (55.27%)	50 (31.85%)		
Yes	663 (47.36%)	556 (44.73%)	107 (68.15%)		
DM				1.71 (1.35;2.08)	0.0042
No	1,105 (78.93%)	995 (80.05%)	110 (70.06%)		
Yes	295 (21.07%)	248 (19.95%)	47 (29.94%)		
COPD				2.95 (2.44;3.47)	<0.0001
No	1,313 (93.79%)	1,178 (94.77%)	135 (85.99%)		
Yes	87 (6.21%)	65 (5.23%)	22 (14.01%)		
Asthma				1.26 (0.65;1.87)	0.45
No	1,304 (93.14%)	1,160 (93.32%)	144 (91.72%)		
Yes	96 (6.86%)	83 (6.68%)	13 (8.28%)		
IHD				2.06 (1.6;2.53)	0.0022
No	1,265 (90.36%)	1,134 (91.23%)	131 (83.44%)		
Yes	135 (9.64%)	109 (8.77%)	26 (16.56%)		
Cirrhosis				0.88 (-1.19;2.95)	0.9
No	1,390 (99.29%)	1,234 (99.28%)	156 (99.36%)		
Yes	10 (0.71%)	9 (0.72%)	1 (0.64%)		
Previous treatment with ACEI, ARB				1.81 (1.48;2.15)	0.00047
No	884 (63.14%)	805 (64.76%)	79 (50.32%)		
Yes	516 (36.86%)	438 (35.24%)	78 (49.68%)		
Previous treatment with NSAID				1.28 (0.84;1.73)	0.27

No	1,200 (85.71%)	1,070 (86.08%)	130 (82.8%)		
Yes	200 (14.29%)	173 (13.92%)	27 (17.2%)		
Previous treatment with montelukast				1.19 (-0.03;2.42)	0.78
No	1,377 (98.36%)	1,223 (98.39%)	154 (98.09%)		
Yes	23 (1.64%)	20 (1.61%)	3 (1.91%)		
Fever 24h				1.09 (0.75;1.42)	0.63
No	801 (57.21%)	714 (57.44%)	87 (55.41%)		
Yes	599 (42.79%)	529 (42.56%)	70 (44.59%)		

Summary of the univariate tests based on the odds ratio yielded by univariate logistic regression models built between every predictor and the mortality response. p-values in bold are $<10^{-6}$. For each numerical predictor, the mean, and the standard deviation (in parentheses) values are indicated. For each categorical predictor, the number, and the percentage (in parentheses) of cases are reported. All variables from Table 1 not appearing in Table 2 are included, with a summary of the univariate tests performed on the complete data set.
