# Supplementary material for: Machine-learning-derived predictive score for early estimation of COVID-19 mortality risk in hospitalized patients
Source: PLoS One. 2022 Sep 22;17(9):e0274171. doi: 10.1371/journal.pone.0274171 (PMC9499271; doi:10.1371/journal.pone.0274171)
Supplement: S2 Table — Summary of the univariate tests based on the odds ratio yielded by univariate logistic regression models built between every predictor and the mortality response. p-values in bold are < 10−6. For each numerical predictor, the mean, and the standard deviation (in parentheses) values are indicated. For each categorical predictor, the number, and the percentage (in parentheses) of cases are reported. All variables from Table 2 are included, with a summary of the univariate tests performed on the calibration data set. (PDF) [file pone.0274171.s005.pdf]

S2 Table. Characteristics of patients in the Calibration data set.

	Total; n = 10,008	Alive; n = 8,354	Deceased; n = 1,654	Odds ratio	*p* value
**Variable**	**m (s.d.) or n (%)**	**m (s.d.) or n (%)**	**m (s.d.) or n (%)**	**(95% C.I.)**	
Age, years	65.87 (15.48)	63.57 (15.21)	77.48 (10.94)	1.08 (1.08;1.09)	**<0.0001**
Oxygen saturation, %	92.75 (5.37)	93.46 (4.7)	89.16 (6.86)	0.88 (0.88;0.89)	**<0.0001**
Platelets, $10^3/mm^3$	206.03 (86.93)	209.17 (87.32)	190.16 (83.14)	1 (1;1)	**<0.0001**
LDH, U/L	365.75 (177.25)	350.9 (157.59)	440.75 (240.74)	1 (1;1)	**<0.0001**
Creatinine, mg/dl	1.07 (0.72)	1 (0.63)	1.4 (0.99)	1.83 (1.75;1.9)	**<0.0001**
Lymphocytes, $10^3/mm^3$	1.47 (3.37)	1.49 (3.32)	1.34 (3.61)	0.98 (0.96;1)	0.094
Leukocytes, $10^3/mm^3$	7.16 (4.56)	6.91 (4.09)	8.45 (6.27)	1.07 (1.06;1.08)	**<0.0001**
Hemoglobin, $10^3/mm^3$	13.71 (2.07)	13.82 (2.01)	13.11 (2.24)	0.84 (0.81;0.87)	**<0.0001**
D dimer, $10^3/mm^3$	1,450.52 (3,148.66)	1,271.49 (2,810.5)	2,354.78 (4,372.96)	1 (1;1)	**<0.0001**
Time init – admission, days	6.98 (5.05)	7.24 (4.98)	5.64 (5.19)	0.92 (0.9;0.93)	**<0.0001**
N of comorbidities	1.34 (1.3)	1.17 (1.22)	2.17 (1.39)	1.7 (1.66;1.74)	**<0.0001**
Altered conscience				4.48 (4.32;4.63)	**<0.0001**
No	9,258 (92.51%)	7,926 (94.88%)	1,332 (80.53%)		
Yes	750 (7.49%)	428 (5.12%)	322 (19.47%)		
Respiratory frequency > 24 bpm				2.97 (2.86;3.08)	**<0.0001**
No	7,879 (78.73%)	6,871 (82.25%)	1,008 (60.94%)		
Yes	2,129 (21.27%)	1,483 (17.75%)	646 (39.06%)		
Cardiac failure				3.78 (3.61;3.94)	**<0.0001**
No	9,344 (93.37%)	7,954 (95.21%)	1,390 (84.04%)		
Yes	664 (6.63%)	400 (4.79%)	264 (15.96%)		
Neurological precedents				2.83 (2.7;2.95)	**<0.0001**
No	8,597 (85.9%)	7,389 (88.45%)	1,208 (73.04%)		
Yes	1,411 (14.1%)	965 (11.55%)	446 (26.96%)		
Neoplasia				2.23 (2.06;2.4)	**<0.0001**
No	9,269 (92.62%)	7,830 (93.73%)	1,439 (87%)		
Yes	739 (7.38%)	524 (6.27%)	215 (13%)		
SBP < 90				2.32 (2.14;2.51)	**<0.0001**
No	9,442 (94.34%)	7,959 (95.27%)	1,483 (89.66%)		
Yes	566 (5.66%)	395 (4.73%)	171 (10.34%)		
Kidney failure				3.55 (3.41;3.69)	**<0.0001**
No	9,032 (90.25%)	7,741 (92.66%)	1,291 (78.05%)		
Yes	976 (9.75%)	613 (7.34%)	363 (21.95%)		

Summary of the univariate tests based on the odds ratio yielded by univariate logistic regression models built between every predictor and the mortality response. p-values in bold are $<10^{-6}$. For each numerical predictor, the mean, and the standard deviation (in parentheses) values are indicated. For each categorical predictor, the number, and the percentage (in parentheses) of cases are reported. All variables from Table 2 are included, with a summary of the univariate tests performed on the calibration data set.
