# Supplementary material for: Machine-learning-derived predictive score for early estimation of COVID-19 mortality risk in hospitalized patients
Source: PLoS One. 2022 Sep 22;17(9):e0274171. doi: 10.1371/journal.pone.0274171 (PMC9499271; doi:10.1371/journal.pone.0274171)
Supplement: S3 Table — Summary of the univariate tests based on the odds ratio yielded by univariate logistic regression models built between every predictor and the mortality response. p-values in bold are < 10−6. For each numerical predictor, the mean, and the standard deviation (in parentheses) values are indicated. For each categorical predictor, the number, and the percentage (in parentheses) of cases are reported. All variables from Table 2 are included, with a summary of the univariate tests performed on the validation data set. (PDF) [file pone.0274171.s006.pdf]

S3 Table. Characteristics of patients in the Validation dataset.

	**Total; n = 2,501**	**Alive; n = 2,092**	**Deceased; n = 409**	**Odds ratio**	***p* value**
**Variable**	**m (s.d.) or n (%)**	**m (s.d.) or n (%)**	**m (s.d.) or n (%)**	**(95% C.I.)**	
Age, years	65.82 (15.31)	63.47 (14.84)	77.85 (11.57)	1.09 (1.08;1.1)	**<0.0001**
Oxygen saturation, %	92.88 (5.12)	93.6 (4.45)	89.22 (6.53)	0.87 (0.85;0.89)	**<0.0001**
Platelets, $10^3/mm^3$	206.06 (89.11)	208.83 (88.38)	191.92 (91.55)	1 (1;1)	0.00047
LDH, U/L	362.84 (175.45)	348.04 (158.21)	438.51 (231.23)	1 (1;1)	**<0.0001**
Creatinine, mg/dl	1.07 (0.72)	0.99 (0.57)	1.48 (1.12)	2.14 (1.99;2.29)	**<0.0001**
Lymphocytes, $10^3/mm^3$	1.44 (2.87)	1.49 (2.96)	1.17 (2.34)	0.94 (0.87;1)	0.049
Leukocytes, $10^3/mm^3$	7.14 (6.15)	6.97 (6.46)	7.97 (4.19)	1.03 (1;1.06)	0.042
Hemoglobin, $10^3/mm^3$	13.66 (2.11)	13.78 (2.03)	13.07 (2.4)	0.86 (0.81;0.91)	**<0.0001**
D dimer, $10^3/mm^3$	1,425.79 (3,509.22)	1,243.31 (3,152.73)	2,359.14 (4,844.83)	1 (1;1)	**<0.0001**
Time init – admission, days	6.83 (4.65)	7.11 (4.5)	5.42 (5.16)	0.9 (0.87;0.93)	**<0.0001**
N of comorbidities	1.35 (1.31)	1.18 (1.23)	2.2 (1.4)	1.72 (1.64;1.8)	**<0.0001**
Altered conscience				4.86 (4.56;5.15)	**<0.0001**
No	2,291 (91.6%)	1,974 (94.36%)	317 (77.51%)		
Yes	210 (8.4%)	118 (5.64%)	92 (22.49%)		
Respiratory frequency > 24 bpm				3.08 (2.86;3.31)	**<0.0001**
No	1,968 (78.69%)	1,722 (82.31%)	246 (60.15%)		
Yes	533 (21.31%)	370 (17.69%)	163 (39.85%)		
Cardiac failure				4.04 (3.71;4.37)	**<0.0001**
No	2,332 (93.24%)	1,992 (95.22%)	340 (83.13%)		
Yes	169 (6.76%)	100 (4.78%)	69 (16.87%)		
Neurological precedents				2.94 (2.68;3.19)	**<0.0001**
No	2,140 (85.57%)	1,846 (88.24%)	294 (71.88%)		
Yes	361 (14.43%)	246 (11.76%)	115 (28.12%)		
Neoplasia				1.99 (1.64;2.33)	**<0.0001**
No	2,314 (92.52%)	1,955 (93.45%)	359 (87.78%)		
Yes	187 (7.48%)	137 (6.55%)	50 (12.22%)		
SBP < 90				1.36 (0.94;1.78)	0.15
No	2,356 (94.2%)	1,977 (94.5%)	379 (92.67%)		
Yes	145 (5.8%)	115 (5.5%)	30 (7.33%)		
Kidney failure				4.06 (3.78;4.35)	**<0.0001**
No	2,261 (90.4%)	1,947 (93.07%)	314 (76.77%)		
Yes	240 (9.6%)	145 (6.93%)	95 (23.23%)		

Summary of the univariate tests based on the odds ratio yielded by univariate logistic regression models built between every predictor and the mortality response. p-values in bold are $<10^{-6}$. For each numerical predictor, the mean, and the standard deviation (in parentheses) values are indicated. For each categorical predictor, the number, and the percentage (in parentheses) of cases are reported. All variables from Table 2 are included, with a summary of the univariate tests performed on the validation data set.
