# Supplementary material for: Machine-learning-derived predictive score for early estimation of COVID-19 mortality risk in hospitalized patients
Source: PLoS One. 2022 Sep 22;17(9):e0274171. doi: 10.1371/journal.pone.0274171 (PMC9499271; doi:10.1371/journal.pone.0274171)
Supplement: S4 Table — The term TP stands for True Positives (TPR for True Positive Rate), TN for True Negatives (TNR for True Negative Rate), FP for False Positives, FN for False Negatives. (PDF) [file pone.0274171.s007.pdf]

**S4 Table**. **Metrics used to evaluate the performance of each classification algorithm**.

| **Metric** | **Expression** |
|---|---|
| Sensitivity (TPR) | $TP/(TP+FN)$ |
| Specificity (TNR) | $TN/(TN+FP)$ |
| AUC | $\int_0^1 TPR(x)dx\ \ , where\ x = 1 - TNR$ |
| Accuracy (Acc) | $(TP\ +\ TN)/(TP+TN+FP+FN)$ |
| F1-score | $2TP/(2TP+FP+FN)$ |
| MCC | $(TP\cdot TN - FP\cdot FN)/\sqrt{(TP+FP)\cdot(TP+FN)\cdot(TN+FP)\cdot(TN+FN)}$ |

TP stands for True Positives (TPR for True Positive Rate), TN for True Negatives (TNR for True Negative Rate), FP for False Positives, FN for False Negatives.
