# Supplementary material for: Machine-learning-derived predictive score for early estimation of COVID-19 mortality risk in hospitalized patients
Source: PLoS One. 2022 Sep 22;17(9):e0274171. doi: 10.1371/journal.pone.0274171 (PMC9499271; doi:10.1371/journal.pone.0274171)
Supplement: S5 Table — The same values are illustrated in S1 Fig. The classifier LR refers to Logistic Regression, PLS-DA to Partial Least Squares—Discriminant Analysis, KPLSDA to Kernel PLS-DA and RF to Random Forest. The parameters correspond to the 2.5% percentile (P2.5), to the 50% percentile (Median) and to the 97.5% percentile (P97.5). (PDF) [file pone.0274171.s008.pdf]

S5 Table. Values of the metrics from S4 Table obtained over the 100 folds of training and testing with the Calibration dataset.

Classifier	Parameter	Specificity	Sensitivity	AUC	Accuracy	F-score	MCC
LR	P2.5	0.7384	0.7957	0.8611	0.7551	0.5255	0.4430
	Median	0.7468	0.8340	0.8640	0.7607	0.5355	0.4518
	P97.5	0.7817	0.8424	0.8684	0.7842	0.5541	0.4665
PLS-DA	P2.5	0.7285	0.7627	0.8428	0.7432	0.5061	0.4187
	Median	0.7612	0.8041	0.8557	0.7670	0.5331	0.4430
	P97.5	0.7788	0.8365	0.8672	0.7820	0.5584	0.4687
KPLSDA	P2.5	0.7530	0.6694	0.8395	0.7486	0.5083	0.4068
	Median	0.7755	0.7729	0.8521	0.7745	0.5396	0.4423
	P97.5	0.8394	0.8297	0.8672	0.7944	0.5784	0.4824
RF	P2.5	0.6861	0.7076	0.8513	0.7158	0.4993	0.4178
	Median	0.7598	0.8129	0.8648	0.7698	0.5397	0.4479
	P97.5	0.8356	0.8711	0.8780	0.8161	0.5738	0.4864

The same values are illustrated in S1 Fig. The classifier LR refers to Logistic Regression, PLS-DA to Partial Least Squares – Discriminant Analysis, KPLSDA to Kernel PLS-DA and RF to Random Forest. The parameters correspond to the 2.5% percentile (P2.5), to the 50% percentile (Median) and to the 97.5% percentile (P97.5).
