# Supplementary material for: Machine-learning-derived predictive score for early estimation of COVID-19 mortality risk in hospitalized patients
Source: PLoS One. 2022 Sep 22;17(9):e0274171. doi: 10.1371/journal.pone.0274171 (PMC9499271; doi:10.1371/journal.pone.0274171)
Supplement: S6 Table — (PDF) [file pone.0274171.s009.pdf]

S6 Table. Percentage of observed mortality at each level of the score for the Calibration and Validation data sets.

Set	Class	Score 0	Score 1	Score 2	Score 3	Score 4	Score 5	Score 6	Score 7	Score 8
Calibration	Alive	97.39	88.39	83.57	75.71	62.51	51.80	41.45	33.33	19.23
	Deceased	2.61	11.61	16.43	24.29	37.49	48.20	58.55	66.67	80.77
Validation	Alive	97.95	91.24	80.32	70.19	66.17	56.93	36.08	24.14	25
	Deceased	2.05	8.76	19.68	29.81	33.83	43.07	63.92	75.86	75
